# Ras oncogene-independent activation of RALB signaling is a targetable mechanism of escape from *NRAS(V12)* oncogene addiction in acute myeloid leukemia

**DOI:** 10.1038/onc.2016.471

**Published:** 2016-12-19

**Authors:** E J Pomeroy, L A Lee, R D W Lee, D K Schirm, N A Temiz, J Ma, T A Gruber, E Diaz-Flores, B S Moriarity, J R Downing, K M Shannon, D A Largaespada, C E Eckfeldt

**Affiliations:** 1 Department of Medicine, Division of Hematology, Oncology and Transplantation, University of Minnesota Medical School, University of Minnesota, Minneapolis, MN, USA; 2 Masonic Cancer Center, University of Minnesota, Minneapolis, MN, USA; 3 Department of Pathology, St Jude Children’s Research Hospital, Memphis, TN, USA; 4 Department of Oncology, St Jude Children’s Research Hospital, Memphis, TN, USA; 5 Department of Pediatrics, University of California, San Francisco, CA, USA; 6 Department of Pediatrics, Division of Hematology and Oncology, Minneapolis, MN, USA; 7 Department of Genetics, Cell Biology, and Development, University of Minnesota, Minneapolis, MN, USA

## Abstract

Somatic mutations that lead to constitutive activation of *NRAS* and *KRAS* proto-oncogenes are among the most common in human cancer and frequently occur in acute myeloid leukemia (AML). An inducible *NRAS(V12)*-driven AML mouse model has established a critical role for continued *NRAS(V12)* expression in leukemia maintenance. In this model genetic suppression of *NRAS(V12)* expression results in rapid leukemia remission, but some mice undergo spontaneous relapse with *NRAS(V12)*-independent (NRI) AMLs providing an opportunity to identify mechanisms that bypass the requirement for Ras oncogene activity and drive leukemia relapse. We found that relapsed NRI AMLs are devoid of *NRAS(V12)* expression and signaling through the major oncogenic Ras effector pathways, phosphatidylinositol-3-kinase and mitogen-activated protein kinase, but express higher levels of an alternate Ras effector, *Ralb*, and exhibit NRI phosphorylation of the RALB effector TBK1, implicating RALB signaling in AML relapse. Functional studies confirmed that inhibiting CDK5-mediated RALB activation with a clinically relevant experimental drug, dinaciclib, led to potent RALB-dependent antileukemic effects in human AML cell lines, induced apoptosis in patient-derived AML samples *in vitro* and led to a 2-log reduction in the leukemic burden in patient-derived xenograft mice. Furthermore, dinaciclib potently suppressed the clonogenic potential of relapsed NRI AMLs *in vitro* and prevented the development of relapsed AML *in vivo*. Our findings demonstrate that Ras oncogene-independent activation of RALB signaling is a therapeutically targetable mechanism of escape from *NRAS* oncogene addiction in AML.

## Introduction

Despite aggressive combination chemotherapy, the majority of patients with acute myeloid leukemia (AML) die of relapsed treatment refractory disease.^1^ Furthermore, a large number of older and/or unfit AML patients cannot tolerate intensive treatment approaches and are cured <10% of the time.^1^ The disappointing outcomes with conventional treatment approaches for AML have driven intense interest in safer and more effective targeted treatment approaches. While the genetic landscape of AML has been extensively characterized, genetically based targeted therapies have yet to be realized and the optimal therapeutic target(s) are not known.^2, 3^

*RAS* proto-oncogenes are mutated in about 10–15% of human AML, and additional recurring AML mutations rely on Ras signaling for their oncogenic effects (that is, *PTPN11*, *NF1*, *FLT3-ITD* and *KIT*).^3, 4, 5, 6^ A critical role for oncogenic *Nras* and *Kras* in leukemogenesis and maintenance of AML cells has been substantiated in genetically engineered mouse models.^7, 8, 9, 10^ Furthermore, the mitogen-activated protein kinase (MAPK) and phosphatidylinositol-3-kinase (PI3K) pathways downstream of Ras have been shown to regulate leukemic stem cell self-renewal in AML.^11^ In fact, the MAPK and PI3K pathways are active in the majority of patient-derived AML samples, further supporting a key role for Ras signaling in AML maintenance.^12, 13^

Oncogenic *RAS* mutations are among the most common molecular alterations in human cancer, and thus Ras has been the focus of intense interest for drug development.^14^ A major obstacle for targeted cancer treatment approaches has been the almost ubiquitous development of treatment resistance. For example, disruption of the post-translational modification of Ras with farnasyltransferase inhibitors demonstrated encouraging preclinical activity, but their clinical activity has been limited owing to resistance conferred by alternative biochemical pathways for the prenylation of Ras.^15^ Targeting BRAF and/or MEK has shown encouraging responses for *BRAF(V600E)* mutant non-small-cell lung cancer and melanoma, but responses have been variable and transient owing to treatment resistance.^16, 17, 18, 19, 20^ It has become clear that diverse mechanisms such as disruption of drug–target interaction, mutations or amplifications that lead to activation of downstream signaling pathways, and/or activation of alternative growth and survival pathways can lead to resistance to most, if not all, targeted cancer therapies.^21, 22^ Therefore, a better understanding of disease and context-specific resistance mechanisms will be essential to develop rational combination strategies for specific diseases.

To model Ras oncogene-targeted therapy for AML, we used a tetracycline-repressible *N**RAS(V12)* and *M**ll-AF9-*driven AML genetically engineered mouse model (tNM AML).^8^ The leukemia cells in this model are *NRAS(V12)*-dependent (NRD), and suppressing *NRAS(V12)* expression leads to rapid remission in leukemic mice, further highlighting the clinical potential of targeting oncogenic *NRAS* in AML.^8^ This model faithfully recapitulates the key challenge for clinically targeted cancer treatment, in that some mice spontaneously relapse with *NRAS(V12)*-independent (NRI) AML, providing a robust tool to study the mechanisms of relapse after Ras oncogene-targeted therapy. We interrogated key cancer signaling pathways, performed global gene expression analysis and performed functional studies to identify mechanisms that drive relapse with NRI AML and provide insight into the rational development of novel targeted treatment approaches for AML.

## Results

### Suppressing Ras oncogene expression in *NRAS(V12)*-driven AML leads to spontaneous relapse with NRI disease

**Figure 1:**
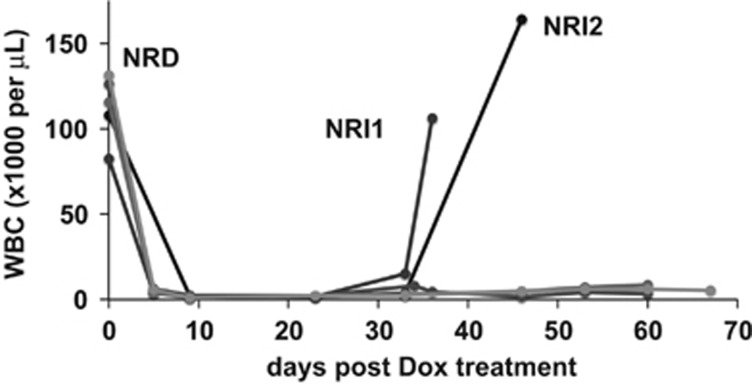
Spontaneous relapse after suppression of Ras oncogene expression in mice with *NRAS(V12)*-driven AML. White blood cell (WBC) counts of leukemic SCID beige mice with *NRAS(V12)*-dependent (NRD) AML rapidly decline after doxycycline (Dox)-mediated suppression of *NRAS(V12)* expression. Two of five mice spontaneously relapsed with NRI AML (NRI1 and NRI1 AMLs) despite continued Dox treatment.

We leveraged the tNM AML model to investigate potential mechanisms of relapse after targeting the Ras oncogene in AML.^8^ In this system, suppression of *NRAS(V12)* expression resulted in rapid leukemia regression (Figure 1). Notably, some mice spontaneously relapsed with NRI AMLs despite continued Dox treatment (Figure 1). Relapsed NRI AMLs were harvested for further characterization (relapsed NRI1 and NRI2 AMLs).

**Figure 2:**
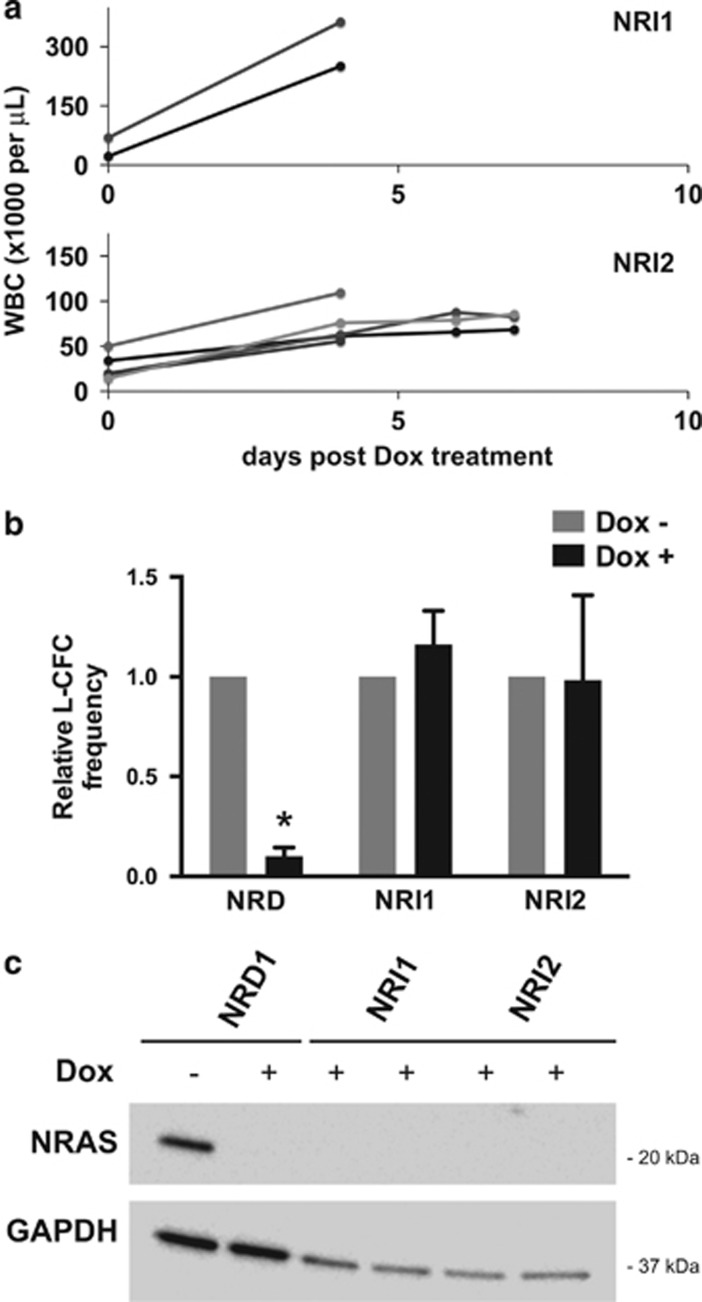
Relapsed NRI AMLs are resistant to doxycycline (Dox)-mediated suppression of *NRAS(V12)* expression and do not re-express oncogenic NRAS protein. (**a**) NRI1 or NRI2 AML cells were transplanted in secondary SCID beige recipient mice. NRI AMLs continued to grow in secondary recipients despite Dox treatment, and mice rapidly succumbed to progressive leukemia. (**b**) NRI1 or NRI2 AML cells harvested from the spleens of leukemic mice were treated *ex vivo* with 1 μg/ml Dox for 48 h and then plated in leukemia colony-forming cell (L-CFC) assays. Results are presented as L-CFC in Dox-treated relative to control treated AML cells (*n*=3 independent experiments, error bars=1 s.d., *P<0.001). (**c**) Western blotting for NRAS protein in splenocytes harvested from mice with NRD and NRI AMLs in the presence or absence of Dox as indicated. NRI AMLs were generated and maintained in the presence of Dox to prevent re-expression of NRAS*(V12)* or re-emergence of NRD AML, so were not evaluated in the absence of Dox.

We confirmed *NRAS* oncogene independence of relapsed NRI AMLs by transplanting NRI1 and NRI2 AMLs into secondary recipients. Unlike the parental *de novo* NRD AML, relapsed NRI1 and NRI2 AMLs did not regress after *in vivo* Dox treatment, and mice rapidly succumbed from progressive leukemia (Figure 2a). Similarly, *ex vivo* treatment of leukemia cells with Dox potently suppressed the clonogenic potential of *de novo* NRD but not relapsed NRI1 or NRI2 AMLs (Figure 2b). Western blotting excluded the possibility of aberrant re-expression of *NRAS(V12)* in relapsed NRI AMLs in the presence of Dox treatment, thereby confirming the *NRAS(V12)* independence of the relapsed NRI AMLs (Figure 2c).

### Analysis of cancer signaling pathways in *de novo* NRD and relapsed NRI AMLs

**Figure 3:**
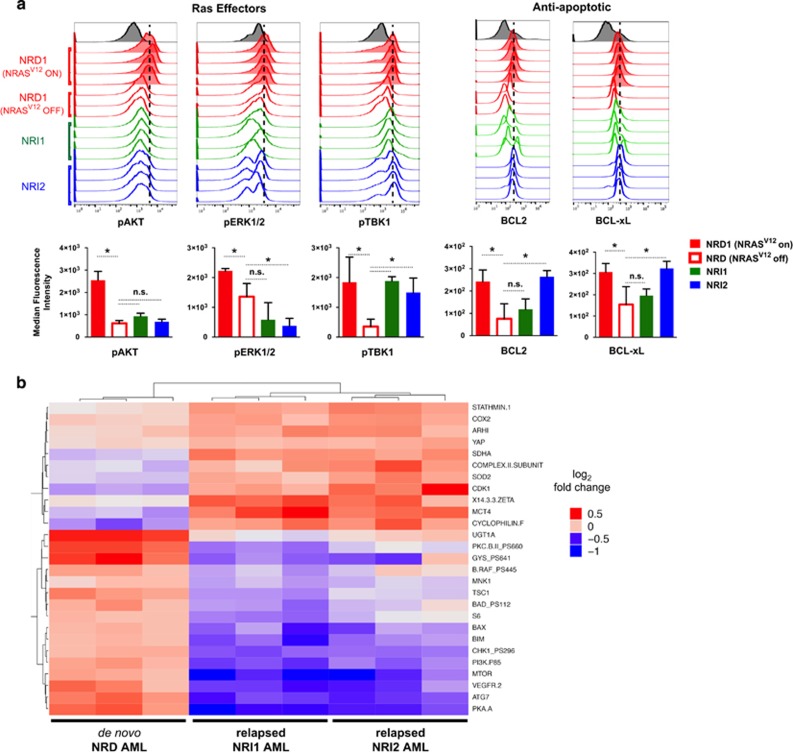
Comparison of canonical Ras effector and cancer signaling pathways in *de novo* NRD AML and relapsed NRI AMLs identifies potential mechanisms that drive relapse with NRI disease. (**a**) Flow cytometry histograms of Ras effector pathways (left) or BCL2 and BCL-xL protein levels (right) in splenocytes from leukemic mice with untreated NRD AML (red shaded), 72 h Dox-treated NRD AML (red open), NRI1 AML (green open) or NRI2 AML (blue open). Signaling through canonical Ras effector pathways was determined by levels of phosphorylated AKT (pAKT) for PI3K, phosphorylated ERK1/2 (pERK1/2) for MAPK and phosphorylated TBK1 (pTBK1) for RALB. Each histogram represents splenocytes from an individual mouse. All mice with NRI AMLs were maintained on Dox to prevent re-expression of *NRAS(V12)*. The median fluorescence intensity for experimental groups is presented below the histograms for each protein of interest (error bars=1 s.d., *P<0.05, NS, nonsignificant *P-*value) (**b**) Heatmap of differential protein levels between Dox-treated *de novo* NRD and relapsed NRI AMLs as determined by RPPA analysis (Benjamini–Hochberg-corrected *q*-value of ⩽0.05).

To investigate potential mechanisms that drive AML relapse after suppressing oncogenic Ras, we performed flow cytometric and reverse-phase protein array (RPPA) analyses of key cancer signaling pathways. Flow cytometric analysis confirmed a decrease in canonical oncogenic Ras effector signaling pathways—MAPK and PI3K—with decreased levels of phosphorylated ERK and AKT following Dox-mediated suppression of *NRAS(V12)* in both *de novo* NRD and relapsed NRI AML cells (Figure 3a). Phosphorylation of TBK1, a key target of RALB signaling, was decreased after Dox-mediated *NRAS(V12)* suppression in *de novo* NRD AML, but was maintained at higher levels in relapsed NRI AMLs, even in the absence of *NRAS(V12)* expression (Figure 3a). The expression of BCL-xL and BCL2 proteins, which are established targets of RALB-TBK1 signaling, were also maintained at higher levels in relapsed NRI AMLs (Figure 3a and Supplementary Figure S1). Proapoptotic proteins BIM, BAD and BAX were expressed at lower levels in NRI AMLs (Supplementary Figure S2), suggesting that an altered balance of pro- and antiapoptotic proteins contributes to the survival of relapsed NRI AML cells. There were no differences in the expression levels or activation of other Ras effector proteins, oncoproteins or tumor suppressor proteins that were evaluated (Supplementary Figure S2).

We complemented our flow cytometric analysis by performing RPPA analysis.^23^ Of the 292 epitopes interrogated, 27 showed different levels of protein expression or phosphorylation between Dox-treated NRD AML and relapsed NRI AMLs (Supplementary Table S1 and Figure 3b). We observed lower levels of Ras-associated proteins including PI3K p85, BRAF(p445), mTOR and PKC βII(p660) in relapsed NRI AMLs compared with NRD AML. Consistent with our flow cytometric analysis, relapsed NRI AMLs also expressed lower levels of proapoptotic mediators BIM and BAX. Relapsed NRI AMLs also had lower levels of CHK1(p296) and increased levels of YAP and CDK1 compared with NRD AMLs. Notably, YAP has been shown to mediate resistance to MAPK targeted therapy and can reverse *Kras* oncogene addiction in a *Kras* oncogene-driven pancreatic cancer model.^24, 25^

### Next-generation RNA sequencing of *de novo* NRD and relapsed NRI AMLs

**Figure 4:**
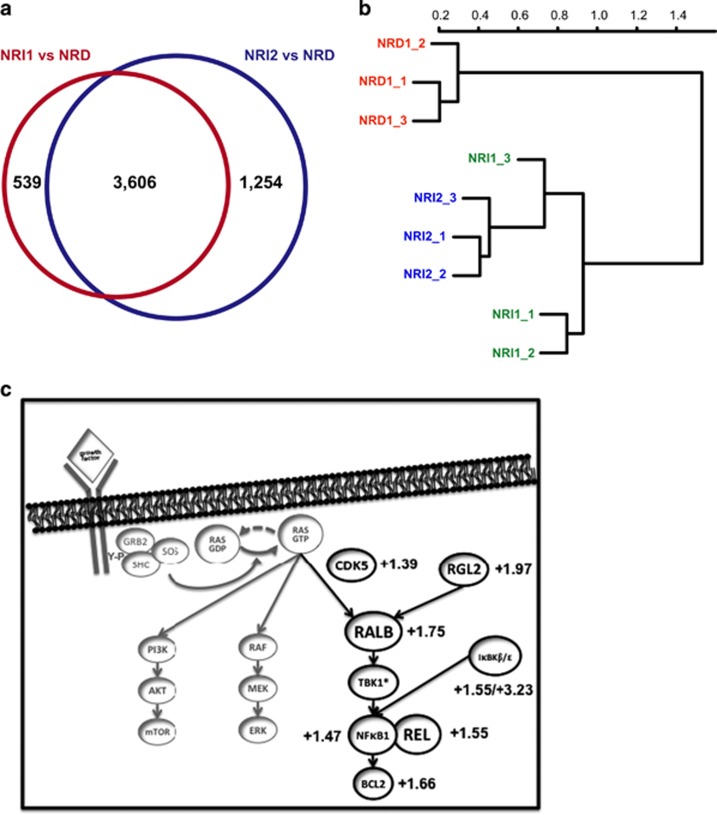
Next-generation sequencing analysis of *de novo* NRD AML and relapsed NRI AMLs. (**a**) Differential transcript levels between splenocytes from leukemic mice with *de novo* NRD AML after 72 h of Dox treatment and relapsed NRI1 and NRI2 AMLs (*n*=3 mice per group, differential expression defined as Benjamini–Hochberg-corrected *q*-value of ⩽0.01- and 1.5-fold change). All mice with NRI AMLs were maintained on Dox to suppress *NRAS(V12)* expression. (**b**) Hierarchical clustering of *de novo* NRD and relapsed NRI1 and NRI2 AMLs. (**c**) Expression of RALB-associated transcripts that are enriched in both relapsed NRI AMLs compared to *de novo* NRD AML. Fold change (NRI/NRD) is indicated and the Benjamini–Hochberg corrected *q*-value is ⩽0.01 for all transcripts.

We then explored potential mechanisms driving the *NRAS* oncogene independence of relapsed NRI AMLs using next-generation RNA sequencing. We interrogated gene expression and transcript characteristics of Dox-treated NRD cells and NRI AMLs. This analysis identified 3606 transcripts that were differentially expressed between both NRD vs NRI1 and NRD vs NRI2 AMLs (Figure 4a). No consistent single-nucleotide variations, copy number alterations by array comparative genomic hybridization or fusion transcripts were identified other than the *Mll-AF9* fusion knock-in gene that is known to be present in both NRD and NRI AMLs (data not shown).^8^ NRI1 and NRI2 AML cells were more similar to each other than NRD AML cells as determined by unsupervised clustering. Notably, NRI1 and NRI2 samples did not segregate in unsupervised clustering based on all identified transcripts (Figure 4b), suggesting that they may share a common mechanism for NRI growth and survival.

We performed Ingenuity Pathway Analysis to identify enriched cellular processes, signaling pathways and predicted upstream regulators of differentially expressed genes (Supplementary Tables S2 and S3). The Tec kinase pathway was among the most activated pathways in relapsed NRI AMLs compared with *de novo* NRD AML (*z*-score 4.333, *P*=5.888 × 10^−6^). Bruton’s tyrosine kinase, a member of the Tec kinase family, has been identified as a targetable pathway in AML.^26, 27, 28^ The PI3K signaling pathway was the most inhibited pathway in relapsed NRI AMLs compared with *de novo* NRD AML (*z*-score=−3.087, *P*=2.570 × 10^−5^), consistent with their decreased dependence on canonical Ras signaling. The canonical nuclear factor-κB signaling pathway was activated in relapsed NRI AMLs compared with *de novo* NRD AML (*z*-score=2.111, *P*=1.479 × 10^−4^) and NFKB1 was among the predicted activated upstream regulators in relapsed NRI AML cells (*z*-score=2.603, *P*=2.000 × 10^−4^). Other predicted upstream regulators that were activated in relapsed NRI AMLs included HOXA9 (*z*-score=2.865, *P*=5.420 × 10^−7^), CEBPA (*z*-score=2.307, *P*=2.510 × 10^−12^) and STAT3 (*z*-score=2.084, *P*=1.340 × 10^−10^) that have established roles in AML.^29, 30, 31^

Many transcriptional regulators were differentially expressed between *de novo* NRD AML and relapsed NRI AMLs (Supplementary Table S4). The leukemogenic transcription factors *Gfi1* and *Myb* were among the most highly upregulated in relapsed NRI AMLs compared with the *de novo* NRD AML. ID1, a transcriptional regulator that cooperates with oncogenic Ras in the development of metastatic breast cancer, was also upregulated in relapsed NRI AMLs.^32^ Similar to flow cytometric and RPPA analyses, several mediators of apoptosis were differentially expressed between *de novo* NRD and relapsed NRI AMLs (Supplementary Figure S3). Notably, relapsed NRI AMLs expressed lower levels of proapoptotic *Bim* and higher levels of antiapoptotic *Bcl2* transcripts.

To further investigate the role of RALB signaling as a mediator of *NRAS(V12)* independence for relapsed NRI AMLs we evaluated the differential expression of genes that are known to be associated with RALB activation and signaling (Figure 4c). Notably, *Cdk5* and *Rgl2*, positive regulators or RALB activation, were both enriched in relapsed NRI AMLs compared with *de novo* NRD AML.^33, 34^ *Ralb* was also upregulated in relapsed NRI AMLs, and RALB effectors including components of the nuclear factor-κB transcriptional complex, *Nfkb1* and *Rel*, and prosurvival *Bcl2* were also enriched in relapse NRI AMLs compared with *de novo* NRD AML. Other noncanonical IκB kinases, IκBKβ and IκBKε, were also upregulated in relapsed NRI AMLs. Taken together, the differential expression of RALB-associated proteins and genes support a key role for RALB signaling in NRI AML relapse.

### Inhibition of RALB activation with dinaciclib has potent antileukemic effects on human AML cells *in vitro*

**Figure 5:**
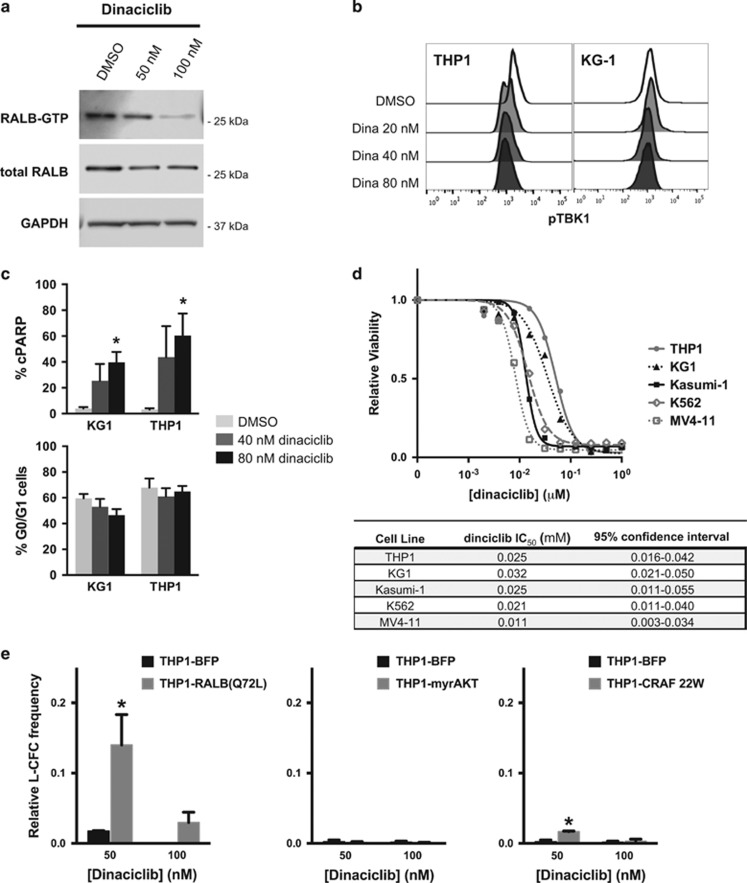
Dinaciclib inhibits RALB activation and has RALB-dependent antileukemic effects in human AML cell lines. (**a**) Western blot for RALB-GTP, total RALB and GAPDH proteins in KG1 AML cells treated for 8 h with DMSO or dinaciclib (representative of three independent experiments). (**b**) Phosphorylated TBK1 (pTBK1) levels in AML cells 24 h after treatment with dinaciclib (representative of three independent experiments). (**c**) Percentage of AML cells with cleaved PARP (cPARP+) (top) and proportion of G_0_/G_1_ cells (bottom) 24 h after treatment with dinaciclib determined by flow cytometry (*n*=3 independent experiments, error bars=s.e.m., *P<0.05). (**d**) MTS viability analysis of AML cell lines 72 h after dinaciclib treatment and calculated half-maximal inhibitory concentrations (IC_50_) (*n*=3–5 independent experiments, error bars are not included for clarity of presentation and are included in Supplementary Figure 4). (**e**) Relative leukemic colony formation (L-CFC) of THP1 transduced with *RALB(Q72L)*, myristoylated *AKT* (*myrAKT*), *CRAF 22W* or control vector (*BFP*) 24 h after treatment with dinaciclib relative to DMSO-treated controls (*n*=3 independent experiments, error bars=1 s.d., *P<0.05).

While clinically relevant direct Ras-like (Ral) inhibitors are lacking, dinaciclib, a cyclin-dependent kinase inhibitor, has been shown to inhibit CDK5-mediated activation of Ral signaling.^33, 35^ We evaluated the effects of dinaciclib on RALB signaling in AML cell lines. *In vitro* treatment of human KG1 AML cells with dinaciclib potently inhibited RALB activation, resulting in a dose-dependent reduction in RALB-GTP levels (Figure 5a). Dinaciclib treatment also led to reduced phosphorylation of the RALB target TBK1 and increased cleavage of apoptotic effector, CASP3 (Figures 5b and c), but did not alter the proportion of cells in the G_0_/G_1_ phase of the cell cycle (Figure 5c). Dinaciclib potently reduced leukemic cell viability with a half-maximal inhibitory concentration in the low nanomolar range for a panel of genetically diverse human AML cell lines (THP1, KG1, Kasumi-1, K562 and MV4-11; Figure 1d and Supplementary Figure S4). To verify the RALB-dependent effects of dinaciclib on AML cells, we rescued the clonogenic potential of THP1 cells by ectopically expressing constitutively activated *RALB(Q72L)*^36^ (Figure 5e). Notably, ectopic expression of constitutively activated forms of other major Ras effectors, myristoylated *AKT* (*myrAKT*) or *CRAF 22W*, did not effectively rescue leukemic colony formation (Figure 5e).

### Dinaciclib induces apoptosis in patient-derived AML samples *in vitro* and has potent antileukemic activity in preclinical AML models *in vivo*

**Figure 6:**
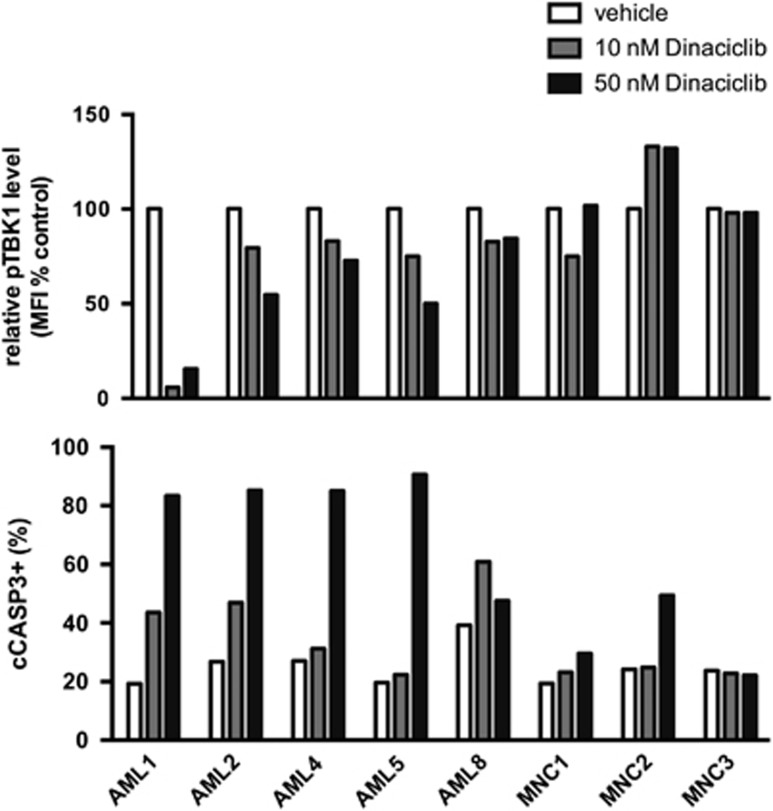
Dinaciclib inhibits RALB-TBK1 signaling and induces apoptosis in primary patient-derived AML cells. (Top) Relative mean fluorescence intensity (MFI) of phosphorylated TBK1 (pTBK1) and (bottom) percentage of cells with cleaved caspase-3 (cCASP3+) in individual AML patient samples (AML, *n*=5) or mononuclear cells from healthy granulocyte-colony stimulating factor (G-CSF) mobilized peripheral blood donors (MPBs, *n*=3) after 24 h treatment with dinaciclib measured by flow cytometry.

We then evaluated effects of dinaciclib on primary patient-derived AML samples. We previously found that primary AML samples have increased levels of RALB-TBK1 signaling compared with normal blood mononuclear cells from healthy mobilized peripheral blood donors.^37^ *In vitro* treatment of a panel of AML samples with dinaciclib uniformly resulted in decreased phosphorylation of TBK1 and increased cleavage of CASP3, while normal mononuclear cells from healthy donors were relatively insensitive to dinaciclib treatment (Figure 6). There was not a clear relationship between the response to treatment and the clinical characteristics of the samples (Supplementary Table S5).

**Figure 7:**
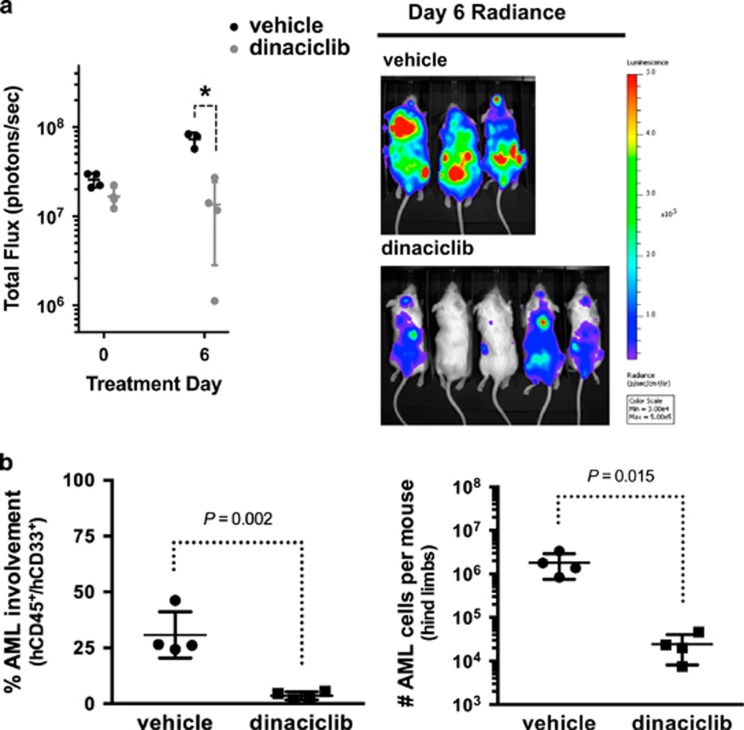
Dinaciclib has potent antileukemic activity in preclinical AML models including patient-derived AML xenograft (PDX) mice. (**a**) Total flux in human THP1-luciferase mouse xenografts treated daily for 5 days with control vehicle or 20 mg/kg dinaciclib (bars=mean±1 s.d., *P<0.05) and radiance images on day 6 after completion of treatment. (**b**) Percentage (left) and absolute number (right) of human CD45 and human CD33-double-positive AML cells in the hindlimb bone marrow from NRGS PDX mice (AML2 sample from Figure 6) after 5 daily treatments with 20 mg/kg dinaciclib or control vehicle (bars=mean±1 s.d.).

To further evaluate the translational potential of our findings, we tested the *in vivo* activity of dinaciclib against human AML cell line mouse xenografts. Mice with established leukemia were treated with five daily doses of 20 mg/kg dinaciclib or control vehicle. A five-day regimen reduced the leukemic burden in human THP1-luciferase mouse xenografts compared with controls (Figure 7a). We then evaluated the activity of dinaciclib against patient-derived AML mouse xenografts (PDX). Leukemic PDX mice were treated with five daily doses of 20 mg/kg dinaciclib or control vehicle. Treatment with dinaciclib led to a marked reduction in bone marrow involvement by human AML cells and a 2-log reduction in absolute leukemic burden compared with control treated PDX mice (Figure 7b).

### Inhibition of RALB activation potently suppresses leukemic colony formation and prevents NRI AML relapse

**Figure 8:**
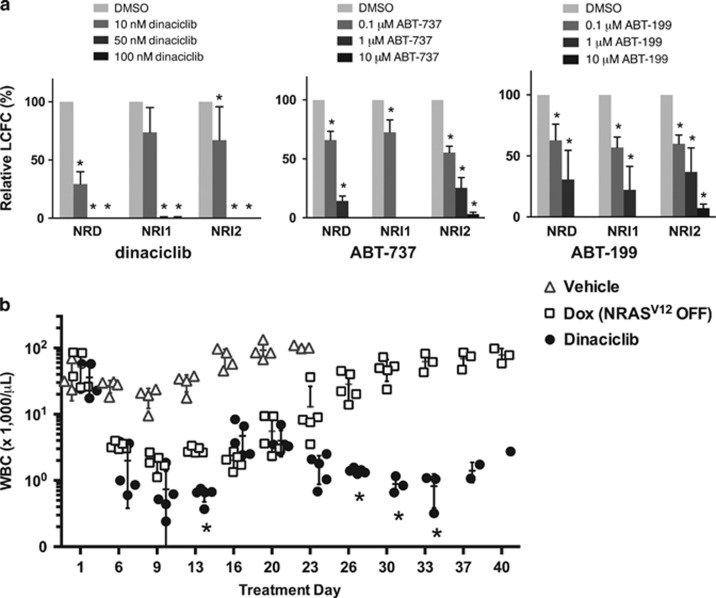
Inhibition of RALB activity with dinaciclib potently suppresses leukemic colony formation *in vitro* and prevents NRI AML relapse *in vivo*. (**a**) Leukemia colony-forming cell (L-CFC) frequencies for splenocytes harvested from leukemic mice treated for 16 h with dinaciclib, ABT-737 or ABT-199 relative to DMSO vehicle controls (*n*=3 independent experiments, error bars=1 s.d., *P<0.05) (**b**) White blood cell counts (WBCs) of leukemic NRG mice transplanted with a 1:1 mix of NRD and NRI1 AML cells treated with control vehicle, continuous doxycycline (Dox) to suppress NRAS(V12) expression or 5 days per week of 15 mg/kg per day dinaciclib (each symbol represents an individual mouse, bars=mean±1 s.d., *P<0.05 between Dox- and dinaciclib-treated mice).

To explore the therapeutic potential for inhibiting RALB activation in relapsed NRI AMLs, we evaluated the activity of dinaciclib against *de novo* NRD and relapsed NRI AMLs. Treatment of *de novo* NRD AML and relapsed NRI AMLs with dinaciclib resulted in a dose-dependent reduction in leukemic colony formation in the low nanomolar range (Figure 8a). Similarly, inhibition of BCL2 family proteins with ABT-737 or ABT-199 suppressed the clonogenic potential of *de novo* NRD AML and relapsed NRI AMLs (Figure 8a), consistent with the established role of BCL2 proteins to support cancer cell survival downstream of RALB.^38^

We then evaluated the *in vivo* activity of dinaciclib in human preclinical AML mouse models. To increase the frequency of AML relapse in the tNM model, we transferred a 1:1 ratio of *de novo* NRD AML and relapsed NRI1 AML cells into recipient mice. Leukemic mice were assigned to treatment with control vehicle, Dox (to suppress *NRAS(V12)* expression), or dinaciclib (Figure 8b). The leukemia initially regressed in Dox-treated mice, but mice relapsed between 3 and 4 weeks despite continued Dox treatment. Conversely, dinaciclib induced a prompt response in leukemic mice, and none of the dinaciclib-treated mice relapsed out to 40 days, further supporting the role of RALB activation in relapse with NRI AML and demonstrating the therapeutic potential of targeting this pathway.

## Discussion

In this study, we investigated potential mechanisms that bypass the requirement for *NRAS(V12)* in AML and represent putative mechanisms of resistance to therapeutically targeting oncogenic NRAS. In the *NRAS(V12)*-addicted tNM AML mouse model, some mice spontaneously relapsed with *NRAS* oncogene-independent AML despite continued suppression of *NRAS(V12)* expression. The spontaneous relapse seen after mimicking *RAS* oncogene-targeted cancer therapy closely resembles the major clinical challenge for targeted cancer therapies in general,^15, 16, 17, 18, 19, 20, 21, 22, 39, 40^ and confirm that *NRAS(V12)*-addicted AML cells can acquire alternative mechanisms for maintained growth and survival and survive in the absence of the *NRAS* oncogene. With renewed efforts to therapeutically target oncogenic Ras proteins, our results have important implications for anticipating therapeutic challenges for such treatment strategies.^41, 42^ Specifically, we found that relapsed AMLs developed an NRI mechanism to maintain signaling through RALB. The ability of inhibition of RALB signaling to suppress the clonogenic potential of relapsed NRI AML and to prevent AML relapse in leukemic mice supports further investigation of this pathway as a therapeutic target. Furthermore, the potent antileukemic effect of inhibiting RALB signaling in human AML, including patient-derived AML cells *in vitro* and *in vivo*, highlights the translational potential for our findings.

There is mounting evidence that the activation and cellular localization of Ral GTPase effector proteins have an important role in Ras-driven transformation, proliferation, migration and survival.^43^ Consistent with this, we recently demonstrated a key role for RALB in AML cell survival.^37^ RALA or RALB activity are essential for cancer cell proliferation in a murine *KRAS*-driven non-small-cell lung cancer model.^44^ While RALB has a central role in innate immune signaling, chronically activated RALB associates with SEC5 in the exocyst complex and activates the noncanonical IκB kinase family member TBK1, a critical mediator of RALB’s oncogenic activity.^45^ The mechanism by which the RALB-TBK1 axis supports cancer cell survival remains unclear and are likely context and disease dependent, but have been shown to involve regulators of normal innate immune signaling in several models.^38, 46^ A large-scale synthetic lethal RNAi screen uncovered a critical role for TBK1 in *KRAS*-driven transformation of epithelial cells through activation of nuclear factor-κB antiapoptotic signals involving REL and BCL-xL.^38^ We found that upregulation of *Cdk5* and *Rgl2*, activators of RALB signaling,^34, 35^ as well as increased *Ralb* expression was associated with NRI activation of RALB-TBK1 signaling in relapsed AMLs. Activation of RALB signaling and upregulation of components of the nuclear factor-κB transcriptional complex were accompanied by an altered balance of pro- and antiapoptotic mediators, consistent with previous reports suggesting that nuclear factor-κB signaling downstream of RALB supports cancer cell survival.^38, 45^ We have previously shown that RALB enhances expression of BCL2 in AML, providing a potential mechanism by which RALB drives NRI AML relapse.^37^ Consistent with this, BCL2 family inhibitors to suppressed the clonogenic potential of relapsed NRI AML cells; however, the precise mechanisms that promote AML cell survival downstream of RALB remain to be elucidated.

While targeting RALB activation with dinaciclib demonstrated potent RALB-dependent antileukemic effects on AML cells, we cannot exclude the contribution of RALB-independent effects. It is possible that dinaciclib-mediated inhibition of CDK1, another protein that was enriched in relapse NRI AMLs, or CDK9, an alternate survival pathway for MLL-rearranged AMLs,^47^ contributes to the antileukemic activity of dinaciclib. In fact, drugs that target multiple pathways may reduce the ability of AML cells to coopt alternate resistance pathways. Our findings support the further investigation of the critical targets of dinaciclib in AML. New small molecules that directly inhibit Ral function may provide additional tools to study the role and therapeutic potential of RALB signaling in AML as they become available.^48^

Our data also identified several other potential mechanisms that may bypass *RAS* oncogene dependence of AML cells. The altered balance of pro- and antiapoptotic mediators in relapsed NRI AML cells that could be a direct or indirect result from enhanced RALB signaling warrants further investigation. YAP, a mediator of the Hippo signaling pathway that has been shown to mediate resistance to inhibition of MAPK signaling and promote *Kras* oncogene independence in oncogenic pancreatic cancer,^24, 25^ was enriched in relapsed NRI AMLs. Overall, the broad deregulation of signaling and gene transcription observed provides a foundation for the ongoing rational development of targeted treatment approaches designed to mitigate or prevent relapse after Ras targeted treatment strategies.

This work provides important insight into the mechanisms of response and resistance to targeted cancer treatment approaches in general, and particularly for renewed efforts to therapeutically target Ras signaling.^41^ Furthermore, our results support further characterization of RALB signaling as a key mediator of survival and *NRAS*-independent relapse in AML and as a valid therapeutic target.

## Materials and methods

### Mouse studies

All mouse studies were approved by the University of Minnesota Institutional Animal Care and Use Committee. Roughly equal number of male and female mice were used for all experiments and evenly allocated to experimental groups without systematic randomization or blinding. Group sizes were determined based on the number of mice required for 80% power to detect a difference in the mean AML burden of at least 1.5 s.d. between experimental and control groups with *P*<0.05 using a t-test. For leukemic cell transplantation, 2 × 10^6^ tNM AML cells, 4 × 10^6^ THP1-luciferase AML cells or 2 × 10^6^ patient-derived AML cells were injected via tail vein into 6–10-week-old recipient mice. Severe combined immunodeficiency (SCID) beige (Charles River, Burlington, MA, USA) mice did not receive any preconditioning before murine AML transplantation. NRG or NRGS (Jackson Labs, Bar Harbor, ME, USA) mice received 375 cGy from an X-ray source 24 h before human AML transplantation. Peripheral blood was obtained by retro-orbital blood sampling and leukocyte counts were monitored using a Hemavet 950 (Drew Scientific, Miami Lakes, FL, USA). THP1-luciferase mice were monitored using an IVIS 100 Imaging System (Perkin-Elmer, Waltham, MA, USA). PDX mice were monitored by flow cytometry of peripheral blood stained for human CD45 and human CD33. Doxycycline-treated mice were given 4 mg intraperitoneally doxycycline (Sigma-Aldrich, Saint Louis, MO, USA) followed by 5 mg/ml in their water.

### Inhibitors

Dinaciclib, ABT-737 and ABT-199 were purchased from Selleck Chemicals (Houston, TX, USA) and reconstituted in dimethyl sulfoxide (DMSO). For *in vitro* studies inhibitors were diluted in the growth medium. For *in vivo* studies dinaciclib was diluted in 20% hydroxypropyl-β-cyclodextrin and administered intraperitoneally.

### Leukemia colony-forming cell assay

NRD and NRI AML cells were cultured in RPMI-1640 (Lonza, Basel, Switzerland) with 10% fetal bovine serum (Atlas Biologicals, Fort Collins, CO, USA) with or without 1 μg/ml doxycycline (Sigma-Aldrich) for 48 h or inhibitors for 24 h and then plated in IMDM (Lonza) with 30% fetal bovine serum, 1.275% methylcellulose (R&D Systems, Minneapolis, MN, USA) and 2 ng/ml murine GM-CSF (R&D Systems). Human AML cells were treated with inhibitors for 24 h and then plated in MethoCult H4034 (Stem Cell Technologies, Vancouver, BC, Canada). Colonies were scored after 7–14 days on an inverted microscope.

### Western blotting and RALB-GTP assay

Protein lysates were run on 10% polyacrylamide gel electrophoresis gels and transferred to a PVDF using the NuPAGE and iBlot Systems (Life Technologies, Carlsbad, CA, USA). RALB-GTP levels were determined using the RALB Activation Assay Kit (EMD Millipore, Billerica, MA, USA). Blots were blocked and stained according to antibody manufacturer’s recommendations. Blots were developed using the SuperSignal West Pico ECL (Thermo Fisher, Minneapolis, MN, USA) or Advansta Quantum ECL Kit (Advansta, Menlo Park, CA, USA) and signals were quantified using the LI-COR Imaging System (LI-COR Biosciences, Lincoln, NE, USA).

### Flow cytometric analysis

Flow cytometric analysis was performed as described previously.^49^ Briefly, for intracellular antigens cells were fixed with 2% paraformaldehyde (Electron Microscopy Sciences, Hatfield, PA, USA) and permeabilized with 90% methanol (Sigma-Aldrich). Cells were stained with antibodies according to the manufacturer’s recommendations. Cells were analyzed on an LSR II or Fortessa Digital Flow Cytometer (BD Biosciences, San Jose, CA, USA) and analyzed using the FlowJo software (Ashland, OR, USA).

### Antibodies

For western blotting, NRAS (F155) mouse monoclonal IgG1 (sc-31) and HRP-conjugated secondary antibodies were purchased from Santa Cruz Biotechnology (Dallas, TX, USA); BCL-xL rabbit polyclonal (2762) and GAPDH (14C10) rabbit monoclonal (2118) were purchased from Cell Signaling Technologies (Danvers, MA, USA); and BCL2 mouse monoclonal (610538) was purchased from BD Biosciences. For flow cytometry, c-MYC (9E10) Alexa Fluor 700, BAD (Y208), BAX (E63), BCL2 (E17), BCL-xL (E18) and BIM (Y36), and MCL1 (Y37) were purchased from Abcam/Epitomics (Cambridge, MA, USA); phospho-4EBP1 (Thr46) and phospho-mTOR (Ser2448) were purchased from Thermo Fisher; cleaved caspase-3 (Asp175, D3E9) phycoerythrin, cleaved PARP (Aps214, 5A1E) Alexa Fluor 647, phospho-4E-BP1 (Thr37/46, 236B4) Alexa Fluor 647, phospho-AKT (Ser473, D9E) Alexa Fluor 488, phospho-p44/p42 MAPK (Erk1/2, Thr202/Tyr204, D13.14.4E) phycoerythrin, BCL-xL (54H6), BIM (C34C5), IkBa (L35A5), PTEN (138G6), phospho-JNK (Thr183/Tyr185, G9), phospho-S6 (Ser235/236) and phospho-TBK1 (Ser172, D52C2) phycoerythrin were purchased from Cell Signaling Technologies; PUMA (RB1353-RB1354) was purchased from Novus Biologicals (Littleton, CO, USA); and phospho-TBK1 (pS172, J133-587) Alexa Fluor 488, cleaved PARP (Asp214), Ki67 (B56), phospho-AKT (pS473, M89-61), phospho-STAT5 (pY694, 47), human CD45 (2D1) FITC and human CD33 (P67.6) phycoerythrin were purchased from BD Biosciences.

### Reverse-phase protein arrays

Total splenocytes were harvested from leukemic mice, snap frozen in liquid nitrogen and sent to the MD Anderson RPPA Core Facility for analysis. Briefly, protein lysates were serially diluted and arrayed on nitrocellulose-coated slides using an Aushon 2470 Arrayer (Aushon BioSystems, Billerica, MA, USA). The slides were scanned, analyzed and quantified using Array-Pro Analyzer (Tecan Group Ltd, Männedorf, Switzerland). Protein concentrations were normalized by median polish, which was corrected across samples by the linear expression values using the median expression levels of all antibody experiments to calculate a loading correction factor for each sample. Normalized protein values were used to evaluate differential target levels between NRD and NRI samples using a Benjamini–Hochberg-corrected *q*-value of ⩽0.05.

### Next-generation RNA sequencing

Total splenocytes were harvested from leukemic mice and RNA was extracted using the RNeasy Midi Kit (Qiagen, Hilden, Germany). RNA samples were quantified, quality checked and analyzed using the Illumina HiSeq 2000 (Illumina, San Diego, CA, USA) at the University of Minnesota Genomics Core Facility. Raw data was mapped to the mouse mm9 genome using TOPHAT2 suite.^50^ Differential expression between NRD and NRI samples was determined using Cuffdiff.^51^ R (R core Team 2013) was used to visualize expression data. Genes with RNAseq expression variation over 0.2 were log transformed and mean centered. The resulting data was clustered using Pvclust package with correlation as distance metric and average clustering method.^52^ Differential transcript expression was defined using a Benjamini–Hochberg-corrected *q*-value of ⩽0.01. The data discussed have been deposited in NCBI's Gene Expression Omnibus (GSE87870).

### Cell culture and primary AML samples

Cell lines were originally obtained from ATCC or DSMZ, maintained under standard cell culture conditions, tested monthly for mycoplasma contamination and authenticated by STR analysis at the University of Arizona Genomics Core. De-identified mobilized peripheral blood and AML patient samples were obtained after informed consent according to protocols approved by the University of Minnesota Institutional Review Board. Primary AML samples were cultured in IMDM (Thermo Fisher) supplemented with 20% fetal bovine serum (Atlas Biologicals, Fort Collins, CO, USA), 100 μm 2-mercaptoethanol (Sigma-Aldrich), 1% GlutaMAX (Thermo Fisher) and 10 ng/ml each of stem cell factor, interleukin-3, interleukin-6, FLT3 (FMS-related tyrosine kinase 3) ligand and thrombopoietin (all from R&D Systems).

### Viable cell enumeration

Viable cell numbers were determined using the CellTiter 96 Aqueous Non-Radioactive Cell Proliferation Assay (Promega, Madison, WI, USA). Half-maximal inhibitory concentrations were calculated using CalcuSyn 2.0 (BioSoft, Cambridge, UK).

### Lentiviral transduction of AML cells

Lentiviral expression vectors were generated using the Gateway cloning system (Thermo Fisher). BFP-expressing lentiviral vectors were generated from a previously described backbone,^53^ by replacing *eGFP* with *eBFP2* and luciferase with *RALB(Q72L)* (from Channing Der via Addgene plasmid no. 19721), *myrAKT* (from John Ohlfest) *or* *CRAF W22* (from Channing Der via Addgene plasmid no. 12593). Vesicular stomatitis virus-G-pseudotyped lentivirus was produced by co-transfecting a 1:2:3 ratio of pMD2.G, pCMVDR8.2 (both from Dider Trono via Addgene nos 12259 and 8455) and lentiviral expression vector into HEK293 cells using X-treme Gene HP (Roche, Basel, Switzerland). Viral supernatant was harvested after 48 h, filtered and used for transduction. Target cells were transduced by coculture with viral supernatant and 5 μg/ml polybrene overnight.

### Statistics

Unless otherwise indicated, statistical differences between two groups were determined using an unpaired, two-tailed *t*-test corrected for multiple comparisons using the Holm–Sidak method with PRISM software (GraphPad, La Jolla, CA, USA). Statistical significance was defined as *P*⩽0.05.
