# Supplementary material for: Ras oncogene-independent activation of RALB signaling is a targetable mechanism of escape from NRAS(V12) oncogene addiction in acute myeloid leukemia
Source: Oncogene. 2016 Dec 19;36(23):3263–73. doi: 10.1038/onc.2016.471 (PMC5464975; doi:10.1038/onc.2016.471)
Supplement: Supplementary Material [file onc2016471x1.pdf]

Supplemental Figure S1

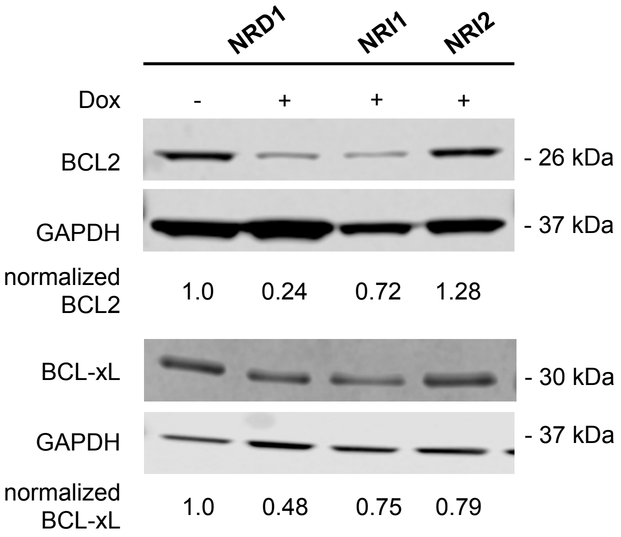

Supplemental Figure S1. Expression of BCL2 and BCL-xL proteins in *de novo* NRD and relapsed NRI AMLs by Western blotting. Western blotting for BCL2 (top) and BCL-xL (bottom) protein levels in splenocytes from leukemic mice with untreated NRD AML, 72 hour Dox treated NRD AML, NRI1 AML, or NRI2 AML. Corresponding GAPDH loading control and GAPDH-normalized quantitation of BCL2 or BCL-xL expression levels are shown.

Supplemental Figure S2

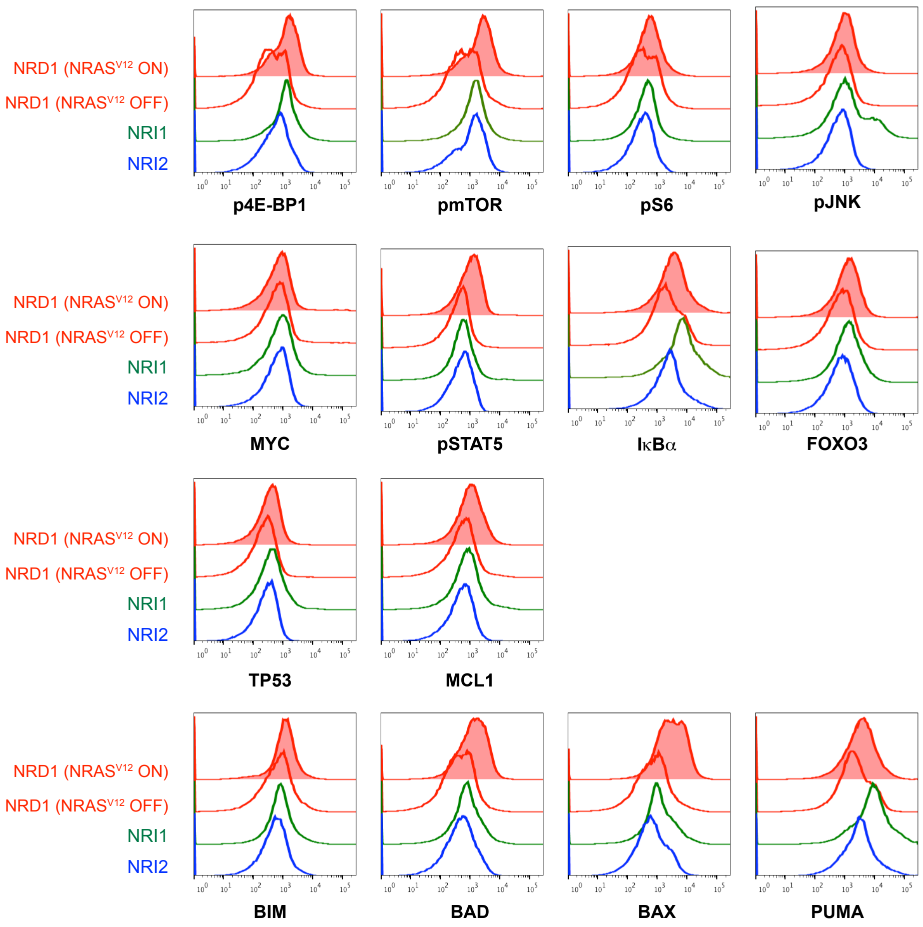

Supplemental Figure S2. Analysis of cancer signaling pathways in *de novo* NRD and relapsed NRI AMLs using flow cytometry. Flow cytometry analysis of signaling through cancer signaling pathways in splenocytes harvested from leukemic mice with untreated NRD AML (red shaded histograms), 72 hour Dox treated NRD AML (red open histograms), NRI1 AML (green open), or NRI2 AML (blue open). All mice with NRI AMLs were maintained on Dox to suppress *NRAS(V12)* expression.

Supplemental Figure S3

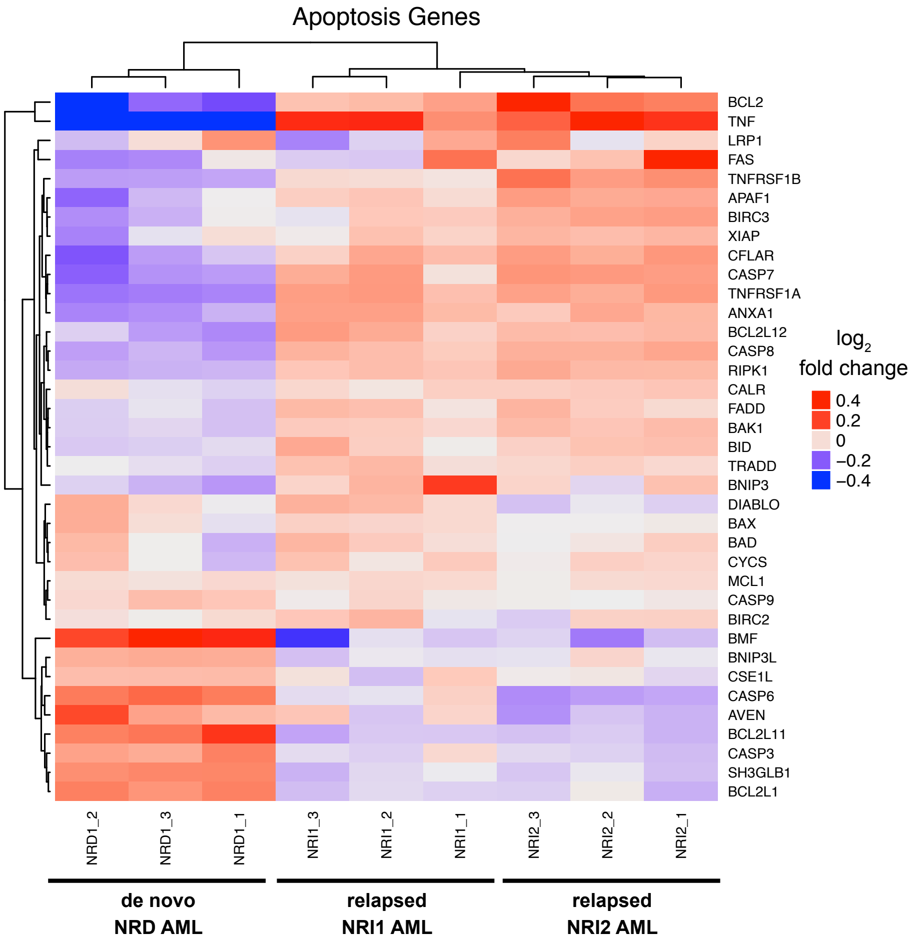

Supplemental Figure S3. Differential expression of apoptosis-associated transcripts between *de novo* NRD and relapsed NRI AMLs. Heatmap for differential apoptosis-associated transcript levels between splenocytes from leukemic mice with *de novo* NRD AML after 72 hours of Dox treatment and relapsed NRI1 and NRI2 AMLs (n = 3 independent mice per group, differential expression defined as Benjamini-Hochberg corrected q-value of ≤ 0.01 and ≥ 1.5 fold change). All mice with NRI AMLs were maintained on Dox to suppress *NRAS(V12)* expression.

Supplemental Figure S4

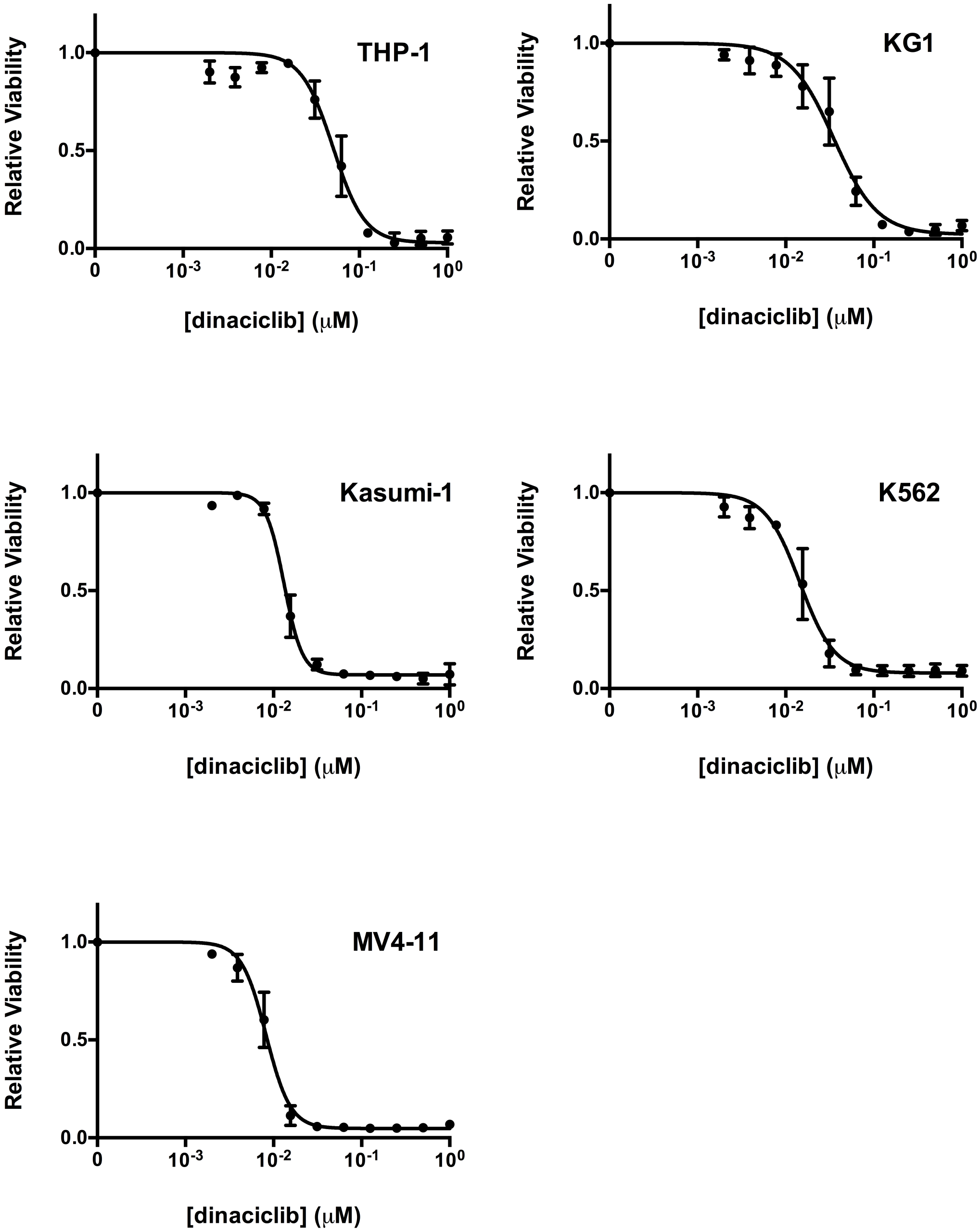

Supplemental Figure S4. Dinaciclib potently reduces viable cell numbers of genetically diverse human AML cell lines. MTS analysis of viable AML cell numbers 72 hours after dinaciclib treatment (n = 3-5 independent experiments, error bars = standard error of the mean).

Table S1. Normalized RPPA Data.

		normalized log2 signal								
Antibody Name	Gene Name	NRD 001	NRD 002	NRD 003	NRI1001	NRI1002	NRI1003	NRI2001	NRI2002	NRI2003
14-3-3-beta	YWHAB	0.000	-0.083	-0.128	-0.096	-0.064	-0.026	0.170	0.289	0.168
14-3-3-epsilon	YWHAE	-0.010	-0.017	0.061	-0.282	-0.275	-0.261	0.009	-0.157	0.000
14-3-3-zeta	YWHAZ	-0.122	-0.072	-0.167	0.583	0.434	0.337	0.464	0.364	0.033
4E-BP1	EIF4EBP1	0.050	0.041	-0.215	-0.955	-0.588	-0.696	-0.323	-0.891	0.000
4E-BP1_pS65	EIF4EBP1	0.000	0.184	-0.530	-0.198	-0.470	0.007	-0.027	-0.247	0.017
4E-BP1_pT37_T46	EIF4EBP1	0.209	0.639	0.000	-0.786	-0.466	-0.042	-0.046	-0.590	-0.685
53BP1	TP53BP1	-0.011	0.080	0.000	0.824	0.299	0.086	0.216	0.503	-0.026
A-Raf	ARAF	-0.022	-0.111	-0.122	0.783	0.572	0.136	0.184	0.400	-0.118
ACC1	ACACA	0.046	-0.011	-0.200	-0.216	-0.028	-0.006	0.164	0.196	0.000
ACC_pS79	ACACA	-0.115	-0.217	-0.745	-0.147	0.122	0.096	0.371	0.397	0.410
ACVRL1	ACVRL1	-0.111	-0.151	-0.124	0.116	0.223	0.136	0.649	0.173	-0.053
ADAR1	ADAR	0.000	-0.096	-0.051	-0.053	-0.240	-0.037	-0.282	-0.192	0.076
Akt	AKT1	0.233	0.277	0.157	0.481	-0.136	-0.257	-0.127	0.160	-0.285
Akt_pS473	AKT1	0.000	-0.217	0.269	0.874	0.257	0.568	0.266	0.553	0.000
Akt_pT308	AKT1	0.000	-0.139	0.037	-0.147	-0.261	-0.384	-0.230	-0.211	-0.253
AMPKa	PRKAA1	0.104	0.008	-0.020	0.033	0.012	-0.037	0.192	0.148	0.000
AMPKa_pT172	PRKAA1	0.103	0.054	-0.189	-0.167	-0.466	-0.535	0.113	0.095	0.000
Annexin-I	ANXA1	0.000	-0.217	0.000	0.874	0.278	0.136	0.266	0.553	0.000
Annexin-VII	ANXA7	0.083	-0.070	-0.181	0.254	0.203	0.136	-0.176	-0.152	-0.242
AR	AR	-0.011	-0.363	0.223	-0.358	0.241	0.136	-0.165	0.309	0.176
ARHI	DIRAS3	-0.013	-0.044	-0.001	0.337	0.121	0.048	0.266	0.267	0.031
ARID1A	ARID1A	0.000	-0.217	0.000	0.874	0.257	0.136	0.266	0.553	0.000
Atg3	ATG3	0.000	0.162	-0.244	-0.023	-0.145	0.131	0.303	0.146	-0.246
Atg7	ATG7	0.480	0.359	0.185	-0.972	-1.332	-1.015	-1.091	-1.197	-0.891
ATM	ATM	0.056	-0.033	0.000	0.874	0.257	0.136	0.266	0.553	0.344
ATM_pS1981	ATM	-0.012	0.048	0.000	-0.059	0.081	-0.108	-0.122	-0.144	0.060
ATP5A	ATP5A	0.000	0.235	-0.131	0.736	0.119	-0.002	0.243	0.415	0.005
ATR_pS428	ATR	0.158	0.081	-0.106	-0.290	0.001	0.006	-0.078	-0.033	0.000
Aurora-B	AIM1	-0.014	-0.080	0.000	0.045	0.162	-0.083	0.055	-0.154	0.154
Axl	AXL	-0.303	0.004	-0.191	0.538	0.198	0.136	-0.070	0.217	0.107
b-Actin	ACTB	0.296	0.130	0.000	-0.381	-0.409	-0.641	-0.087	-0.016	-0.731
b-Catenin	CTNNB1	0.000	-0.217	0.000	0.874	0.257	0.136	0.266	0.553	0.000
b-Catenin_pT41_S45	CTNNB1	0.135	-0.053	0.000	0.017	-0.126	0.026	-0.381	-0.419	-0.196
B-Raf	BRAF	0.000	-0.250	0.054	-0.150	0.094	0.094	-0.054	0.108	0.011
B-Raf_pS445	BRAF	0.206	0.202	0.000	-0.530	-0.513	-0.354	-0.025	-0.317	-0.125
B7-H3	CD276	0.000	0.216	-0.313	0.228	-0.172	0.186	-0.119	-0.115	-0.190
B7-H4	VTCN1	-0.020	0.052	0.000	-0.397	-0.081	0.044	0.053	-0.077	0.171
Bad_pS112	BAD	0.295	0.132	0.000	-0.754	-0.535	-0.587	-0.333	-0.363	-0.116
Bak	BAK1	0.000	-0.062	-0.054	-0.076	-0.040	-0.071	0.037	0.161	0.041
BAP1	BAP1	0.189	0.092	-0.305	-0.176	-0.233	-0.231	-0.154	-0.020	0.293
Bax	BAX	0.128	0.094	0.000	-1.196	-0.879	-0.534	-0.450	-1.016	-0.564
Bcl-xL	BCL2L1	-0.068	-0.027	-0.080	-0.165	0.177	0.055	0.266	0.066	0.042
Bcl2	BCL2	-0.278	-0.007	0.027	0.000	0.155	0.061	-0.033	-0.443	0.000
Bcl2A1	BCL2A1	-0.025	-0.204	0.000	-0.041	-0.021	-0.041	0.028	0.002	0.074
Beclin	BECN1	0.000	-0.101	-0.237	-0.921	-0.277	-0.290	0.011	-0.108	0.050
Bid	BID	0.026	0.186	0.000	-0.330	-0.328	-0.259	-0.113	-0.318	-0.106
Bim	BCL2L11	0.112	0.078	0.000	-1.317	-1.028	-0.804	-0.601	-0.752	-0.511
BRD4	BRD4	-0.122	0.179	0.049	0.281	0.338	-0.151	-0.273	-0.040	-0.278
c-Abl	ABL1	0.023	-0.089	-0.116	-0.174	-0.007	-0.082	0.112	0.101	0.000
c-Jun_pS73	JUN	0.000	0.101	-0.183	0.063	0.132	0.045	0.007	0.036	0.029
c-Kit	KIT	-0.043	-0.211	0.086	-0.173	0.058	0.031	-0.269	-0.193	0.000
c-Met_pY1234_Y1235	MET	0.000	-0.173	-0.010	-0.066	0.051	0.065	0.068	0.113	0.142
c-Myc	MYC	0.000	0.020	-0.118	0.038	-0.030	0.073	0.231	0.346	0.126
C-Raf	RAF1	0.000	-0.044	-0.052	-0.150	-0.423	-0.457	-0.341	-0.233	0.008
C-Raf_pS338	RAF1	0.019	-0.095	0.000	0.071	0.060	-0.008	0.111	-0.074	-0.044
Caspase-3	CASP3	-0.096	-0.181	0.000	-0.184	0.072	0.020	0.108	0.132	0.086
Caspase-7-cleaved	CASP7	0.000	-0.023	0.248	0.422	0.296	-0.234	0.179	0.643	0.233
Caveolin-1	CAV1	-0.213	-0.050	0.000	0.482	0.322	-0.037	-0.015	0.161	-0.027
CD171	L1CAM	0.101	0.159	0.071	0.255	0.257	0.018	-0.242	-0.095	-0.029
CD26	DPP4	0.112	0.007	0.000	-0.247	-0.168	-0.187	0.105	0.416	-0.065
CD29	CD29	-0.159	0.166	-0.123	0.049	0.257	0.242	-0.072	-0.062	-0.064
CD31	PECAM1	0.000	-0.046	-0.049	-0.131	0.043	0.087	-0.213	-0.189	0.137
CD44	CD44	0.246	0.292	-0.076	0.117	-0.001	0.136	-0.171	-0.056	0.146
CD49b	ITGA2	0.000	-0.133	-0.004	-0.062	-0.061	-0.125	0.236	-0.004	0.028
cdc25C	CDC25C	-0.149	-0.156	-0.905	-0.685	-0.466	0.136	-0.274	0.184	0.356
CDK1	CDK1	-0.387	-0.488	-0.555	0.121	0.087	0.136	0.289	0.419	0.831
Chk1	CHEK1	0.146	-0.140	-0.171	0.479	0.075	0.022	-0.129	0.158	0.000
Chk1_pS296	CHEK1	0.145	0.076	0.082	-0.722	-0.931	-1.051	-0.769	-0.719	-0.708
Chk1_pS345	CHEK1	0.062	-0.142	-0.100	-0.385	-0.366	-0.113	-0.082	-0.239	0.000
Chk2	CHEK2	0.188	0.354	0.000	0.229	-0.019	0.051	0.109	-0.126	-0.008
Chk2_pT68	CHEK2	-0.106	-0.061	-0.243	0.110	0.275	0.136	-0.203	-0.043	-0.027
Claudin-7	CLDN7	-0.005	0.161	0.104	0.010	-0.123	0.018	-0.318	-0.177	0.000
COG3	COG3	0.000	0.089	-0.050	0.127	-0.058	-0.095	-0.050	-0.154	0.122
Collagen-VI	COL6A1	0.022	-0.157	-0.119	0.682	0.097	0.030	0.337	0.458	-0.103
Complex-II-Subunit	SDHB	-0.175	-0.217	-0.445	0.283	0.037	-0.111	0.517	0.285	0.165
Connexin-43	CNST43	0.057	0.070	0.028	0.422	0.184	-0.167	-0.187	0.101	-0.319
Coup-TFII	NR2F2	0.000	0.120	0.032	-0.003	0.172	0.000	0.028	0.135	-0.009
Cox-IV	PTGS3	-0.038	-0.166	-0.094	0.065	0.106	0.046	0.266	0.232	0.037

Cox2	CMC2	-0.003	0.027	-0.089	0.072	0.122	0.136	0.228	0.234	0.099
CXCR4	CXCR4	0.049	0.010	0.000	0.189	-0.102	-0.147	0.009	0.189	-0.162
Cyclin-B1	CCNB1	0.000	-0.217	0.021	0.874	0.257	0.136	0.266	0.553	0.040
Cyclin-D1	CCND1	-0.004	0.028	0.000	0.130	0.142	0.116	0.207	-0.086	0.130
Cyclin-E1	CCNE1	0.183	0.321	-0.003	-0.031	-0.006	-0.008	0.086	0.269	0.000
Cyclophilin-F	PPIF	-0.899	-0.510	-0.565	0.442	0.102	0.136	0.486	0.231	0.125
D-a-Tubulin	TUBA1A	0.000	0.014	-0.158	0.143	0.169	0.060	0.283	0.167	-0.024
DJ1	PARK7	0.149	0.021	0.000	-0.116	-0.088	-0.203	-0.201	-0.140	-0.218
DM-Histone-H3	HISTH3	0.008	-0.061	-0.217	-0.087	-0.087	-0.036	0.266	0.177	-0.046
DM-K9-Histone-H3	H3K9ME2	0.024	-0.109	-0.247	-0.108	0.015	0.057	0.198	0.139	0.000
DUSP4	DUSP4	-0.004	-0.009	-0.205	0.036	0.222	0.136	-0.037	-0.072	0.036
Dvl3	DVL3	0.265	0.096	0.000	-0.554	-0.363	-0.301	-0.088	-0.227	-0.169
E-Cadherin	CDH1	0.601	0.363	-0.151	0.567	-0.049	0.367	-0.014	0.246	0.000
E2F1	E2F1	0.044	-0.080	-0.041	-0.149	0.145	-0.040	-0.228	-0.072	0.000
eEF2	EEF2	0.037	-0.003	-0.078	-0.179	0.165	0.136	0.115	-0.067	-0.150
eEF2K	EEF2K	0.000	-0.027	0.000	0.874	0.257	0.136	0.266	0.553	0.000
EGFR	EGFR	-0.107	0.080	-0.071	0.248	-0.150	0.136	0.169	-0.051	0.255
EGFR_pY1068	EGFR	-0.695	0.145	-0.302	-0.231	-0.074	0.136	0.271	-0.467	-0.152
EGFR_pY1173	EGFR	0.000	-0.159	-0.003	-0.246	-0.229	-0.221	0.115	0.124	0.042
eIF4E	EIF4E	-0.023	-0.073	-0.112	1.480	1.159	0.801	0.347	0.335	0.000
eIF4G	EIF4G1	0.000	-0.217	0.024	0.874	0.257	0.136	0.266	0.553	0.000
Elk1_pS383	ELK1	-0.097	-0.152	-0.082	-0.273	-0.114	0.314	-0.139	-0.321	0.000
EMA	EMA	0.007	-0.217	0.159	-0.099	0.339	-0.152	-0.155	-0.370	-0.024
ER	ESR1	0.085	0.054	-0.086	-0.165	-0.001	0.136	0.076	-0.211	-0.047
ERCC1	ERCC1	0.062	-0.057	-0.040	-0.128	-0.022	-0.147	0.144	0.249	0.000
ERCC5	ERCC5	0.000	0.296	0.296	0.477	-0.044	-0.061	0.036	0.156	-0.128
Ets-1	ETS1	-0.092	-0.168	0.032	-0.357	-0.237	-0.333	0.009	0.068	0.000
FAK	PTK2	-0.095	-0.217	0.050	0.183	0.079	-0.112	-0.117	-0.187	0.046
FAK_pY397	PTK2	-0.331	0.096	-0.100	-0.017	0.128	0.301	0.191	-0.426	0.000
FASN	FASN	0.000	-0.171	-0.018	-0.028	0.219	-0.021	-0.287	-0.136	0.039
Fibronectin	FN1	-0.077	1.293	-0.116	-0.694	-0.103	-0.703	1.940	0.553	-1.346
FoxM1	FOXM1	-0.134	0.176	-0.032	0.192	-0.158	0.039	0.266	0.243	0.413
FoxO3a	FOX3	0.272	0.105	0.000	-0.374	-0.129	0.013	-0.215	-0.391	-0.397
FoxO3a_pS318_S321	FOXO3	0.080	0.051	0.000	-0.105	-0.184	0.005	-0.279	-0.241	-0.049
FRA-1	FOSL1	-0.005	0.347	0.000	0.534	0.115	0.127	0.129	0.357	0.002
G6PD	G6PD	0.000	0.055	-0.009	0.154	0.084	-0.059	0.221	0.323	0.149
Gab2	GAB2	0.066	-0.068	0.000	0.485	-0.096	-0.084	0.025	0.164	-0.245
GAPDH	GAPDH	0.000	0.378	0.089	0.004	0.150	0.085	-0.466	-0.383	-0.015
GATA3	GATA3	0.000	0.000	0.010	-0.008	0.106	0.000	-0.185	-0.058	-0.039
GCN5L2	KAT2A	-0.246	-0.128	-0.385	-0.286	0.044	0.181	0.266	-0.009	-0.171
Glutamate-D1-2	GLUD	-0.124	0.353	0.051	-0.573	-0.344	-0.293	-0.500	-0.397	0.000
Glutaminase	GLS	0.064	-0.174	0.000	-0.092	-0.316	-0.028	-0.417	-0.383	-0.320
GPBB	PYGM	0.155	0.139	0.000	-0.080	-0.085	-0.155	0.055	-0.075	-0.121
GSK-3a-b	GSK3A/GSK3B	0.081	0.177	0.330	-0.070	-0.013	-0.052	0.193	0.396	0.000
GSK-3a-b_pS21_S9	GSK3A GSK3B	0.455	0.288	0.000	0.522	-0.095	-0.050	-0.086	0.201	-0.352
Gys	GYS1	0.105	0.063	-0.192	0.881	0.395	0.447	0.169	0.146	0.038
Gys_pS641	GYS1	0.788	0.613	0.339	-0.765	-0.889	-0.628	-1.120	-0.907	0.000
H2AX_pS140	H2AFX	0.000	-0.074	0.014	-0.167	-0.087	-0.097	-0.108	-0.070	-0.142
HER2	ERBB2	0.000	0.055	0.000	0.874	0.257	0.170	0.266	0.553	0.000
HER2_pY1248	ERBB2	-0.088	0.345	-0.120	0.147	0.132	0.180	-0.062	-0.198	0.000
HER3	ERBB3	0.000	-0.014	-0.054	0.126	-0.263	0.152	0.083	0.125	-0.124
HER3_pY1289	ERBB3	0.094	0.019	0.000	-0.274	-0.179	-0.071	-0.231	-0.173	-0.009
Heregulin	NRG1	-0.011	-0.064	-0.009	0.069	0.257	-0.010	0.247	0.163	0.181
HES1	HES1	0.000	-0.098	-0.744	-0.043	-0.376	0.004	-0.578	-0.407	0.143
Hexokinase-II	HK2	-0.186	0.173	-0.215	0.288	0.138	0.136	0.128	0.086	0.012
HIAP	BIRC2	0.022	-0.059	-0.017	-0.573	-0.354	-0.514	-0.509	-0.525	0.000
Hif-1-alpha	HIF1A	-0.140	-0.116	-0.020	0.129	0.204	0.199	-0.576	-0.365	0.000
Histone-H3	HIST3H3	0.086	0.165	-0.380	-0.285	0.257	0.149	0.234	-0.164	-0.116
HSP27	HSBP1	0.187	0.243	-0.155	-0.125	-0.176	-0.149	0.130	0.029	0.000
HSP27_pS82	HSBP1	-0.038	-0.204	0.000	-0.596	0.260	0.053	-0.203	0.013	-0.443
HSP70	HSPA1A	0.058	0.067	-0.046	-0.141	0.257	0.094	-0.030	-0.250	-0.057
IGF1R_pY1135_Y1136	IGF1R	0.000	-0.020	-0.025	-0.085	-0.012	0.013	-0.037	-0.024	0.126
IGFBP2	IGFBP2	0.115	0.067	0.000	0.106	0.122	0.036	0.090	0.238	-0.123
IGFBP5	IGFBP5	0.000	-0.079	-0.186	-0.551	0.023	-0.028	-0.104	0.120	0.224
IGFRb	IGF1R	-0.040	0.226	-0.016	0.051	0.071	0.136	-0.014	-0.143	0.012
INPP4b	INPP4B	0.127	0.114	-0.026	-0.072	-0.238	-0.185	-0.182	-0.221	0.016
IRF-1	IRF1	-0.066	-0.122	-0.444	-0.333	-0.074	0.136	-0.082	-0.616	0.050
IRS1	IRS1	-0.067	-0.069	0.000	0.161	-0.067	-0.078	-0.117	0.068	0.092
JAB1	JAB1	0.016	0.245	0.000	0.486	0.106	0.008	-0.125	0.168	-0.015
Jagged1	JAG1	-0.074	-0.149	0.000	-0.499	-0.274	-0.350	-0.593	-0.262	0.003
Jak2	JAK2	0.006	-0.185	0.000	-0.894	-0.525	-0.233	-0.725	-1.032	-0.239
JNK2	MAPK9	0.231	0.182	-0.092	-0.512	-0.430	-0.130	-0.210	-0.379	0.000
JNK_pT183_Y185	MAPK8	-0.031	-0.101	0.000	0.102	0.165	0.102	0.097	0.140	0.018
LC3A-B	LC3AB	-0.118	0.002	-0.188	0.078	0.102	0.051	0.266	0.099	0.037
Lck	LCK	0.407	0.302	0.072	0.181	-0.138	-0.091	-0.163	0.009	-0.079
LDHA	LDHA	-0.190	0.224	0.342	0.581	0.199	0.000	-0.027	0.260	0.000
MAPK_pT202_Y204	MAPK3	-0.018	-0.025	-0.096	0.034	0.129	0.136	-0.051	0.065	0.101
Mcl-1	MCL1	0.052	0.147	-0.010	0.277	0.178	0.083	0.215	0.225	0.000
MCT4	SLC16A4	-0.257	-0.319	-0.606	0.788	0.257	0.516	0.388	0.331	0.420

MDM2_pS166	MDM2	0.049	-0.100	0.000	0.023	-0.019	0.091	0.110	-0.297	-0.058
MEK1	MAP2K1	0.000	-0.091	0.000	0.874	0.257	0.136	0.266	0.553	0.000
MEK1_pS217_S221	MAP2K1	-0.014	-0.104	0.066	-0.036	-0.001	-0.047	-0.211	-0.154	0.000
MEK2	MAP2K2	0.000	0.249	-0.037	0.254	0.238	0.131	0.241	0.280	0.424
Merlin	NF2	-0.006	-0.011	0.000	-0.067	-0.417	-0.536	-0.565	-0.388	0.058
MIF	MIF	0.000	0.018	-0.440	-0.425	-0.365	0.047	0.190	0.043	0.361
MIG6	ERRFI1	-0.035	-0.053	0.000	-0.069	-0.062	-0.122	0.086	0.057	0.205
Mitochondria	MTCO2	0.124	0.161	0.000	0.524	0.176	0.099	0.018	0.203	-0.069
MMP2	MMP2	0.007	0.132	0.000	-0.226	0.021	-0.198	0.118	0.085	-0.030
Mnk1	MKNK1	0.077	-0.036	0.007	-0.470	-0.526	-0.406	-0.271	-0.400	-0.344
MSH2	MSH2	0.055	0.131	-0.048	0.140	-0.013	0.136	-0.199	0.181	0.147
MSH6	MSH6	-0.079	0.027	0.000	0.395	-0.065	0.046	-0.025	0.221	0.137
mTOR	MTOR	0.163	0.204	0.000	-1.416	-1.800	-1.260	-1.192	-1.580	-0.678
mTOR_pS2448	MTOR	0.096	0.246	0.000	-0.215	0.087	-0.007	0.123	0.251	-0.042
Myosin-11	MYH11	0.000	0.378	0.321	0.193	0.175	-0.415	0.001	0.372	-0.013
Myosin-IIa_pS1943	MYO2A	0.000	-0.217	0.000	0.874	0.257	0.136	0.266	0.553	0.000
Myt1	MYT1	0.058	0.061	-0.109	0.140	0.222	0.136	0.068	-0.294	-0.048
N-Cadherin	CDH2	0.000	0.124	-0.100	0.048	-0.015	0.002	0.049	-0.212	0.293
N-Ras	NRAS	0.021	-0.091	-0.079	-0.042	-0.116	-0.100	0.235	0.139	0.000
NAPSIN-A	NAPSA	-0.016	-0.217	0.011	-0.108	0.027	-0.020	-0.049	0.237	0.602
NDRG1_pT346	NDRG1	0.076	-0.120	0.000	0.527	-0.089	-0.211	-0.081	0.206	-0.347
NDUFB4	NDUFB4	0.000	0.068	-0.209	0.013	0.023	-0.086	-0.076	0.037	0.104
NF-kB-p65_pS536	NFKB1	0.000	-0.073	-0.027	-0.301	-0.314	-0.160	-0.102	-0.079	0.043
Notch1	NOTCH1	-0.014	0.154	0.000	0.075	0.185	0.225	0.007	-0.206	-0.057
Notch3	NOTCH3	0.055	0.096	-0.079	0.087	-0.067	-0.047	0.112	0.103	0.000
P-Cadherin	CDH3	-0.015	-0.014	0.079	0.065	0.099	0.136	-0.302	-0.434	-0.257
p16INK4a	CDKN2A	0.091	0.121	0.000	-0.557	-0.285	-0.570	0.153	-0.285	-0.031
p21	CDKN1A	0.232	0.305	0.000	0.116	-0.011	0.006	0.163	-0.146	-0.027
p27-Kip-1	CDKN1B	-0.033	-0.217	0.017	-0.167	0.037	-0.127	-0.273	0.104	0.018
p27_pT157	CDKN1B	0.033	-0.130	-0.061	0.060	0.163	0.049	0.123	-0.104	0.000
p27_pT198	CDKN1B	0.082	-0.021	0.000	0.116	0.134	0.060	0.139	0.212	-0.051
p38-MAPK	MAPK14	0.450	0.588	0.457	-0.694	-1.859	-0.674	0.038	0.391	0.000
p38_pT180_Y182	MAPK14	0.848	0.102	-0.355	0.844	-0.600	-0.851	-0.123	0.553	0.455
p44-42-MAPK	MAPK3	0.000	0.080	0.323	0.486	-0.131	-0.158	0.479	0.165	-0.388
p53	TP53	0.226	0.070	-0.089	-0.293	-0.501	0.136	0.077	-0.250	-0.218
p70-S6K1	RPS6KB1	0.000	-0.047	0.670	0.874	0.358	0.136	0.266	0.553	0.000
p70-S6K_pT389	RPS6KB1	0.019	-0.173	0.239	-1.077	-0.288	-0.326	-0.331	-0.574	-0.210
p90RSK_pT573	RPS6K	0.153	0.014	-0.212	-0.023	-0.306	0.136	-0.282	-0.499	-0.140
PAI-1	SERPINE1	0.056	0.095	-0.042	0.061	0.070	0.089	0.013	-0.237	0.000
PAR	PAR	0.000	-0.040	0.109	-0.038	-0.014	-0.426	0.279	-0.165	0.089
PARP1	PARP1	0.000	0.387	0.331	0.007	-0.023	0.065	0.185	0.248	-0.064
Paxillin	PXN	0.000	-0.049	0.055	0.874	0.318	0.197	0.266	0.553	0.000
PCNA	PCNA	0.120	-0.056	0.000	-0.133	-0.117	-0.239	0.189	-0.317	-0.013
PD-L1	CD274	0.000	0.211	-0.052	0.042	0.082	-0.098	-0.141	-0.017	0.174
Pdcd-1L1	PDCD1	0.022	-0.048	-0.100	-0.657	0.063	-0.064	0.128	-0.239	0.000
Pdcd4	PDCD4	0.358	0.044	-0.123	-0.244	0.102	0.136	0.126	0.066	-0.208
PDGFR-b	PDGFR	-0.032	0.352	0.498	0.142	0.179	-0.637	0.577	0.375	-0.473
PDK1	PDK1	-0.012	0.197	-0.029	-0.050	0.091	0.136	0.140	0.152	1.373
PDK1_pS241	PDK1	0.177	0.041	-0.008	-0.049	-0.169	0.043	0.179	0.142	0.000
PEA-15	PEA15	0.519	0.441	0.000	-0.748	-0.708	-0.776	-0.447	-0.358	-0.195
PEA-15_pS116	PEA15	0.000	-0.130	-0.154	0.027	-0.021	-0.133	0.126	0.211	0.061
PI3K-p110-a	PIK3C2A	0.185	-0.081	0.000	-0.493	-0.606	-0.525	-0.208	-0.208	-0.108
PI3K-p110-b	PIK3BC	0.020	-0.074	-0.020	0.035	0.117	-0.072	0.176	-0.088	0.000
PI3K-p85	PIK3R1	0.254	0.184	0.037	-1.056	-1.094	-0.978	-0.892	-0.620	-0.598
PKA-a	PRKAR1A	0.522	0.444	0.164	-1.285	-1.610	-1.420	-1.235	-1.203	-0.946
PKC-a	PRKCA	0.096	0.017	0.000	0.163	-0.129	-0.054	-0.295	0.108	-0.102
PKC-a_pS657	PRKCA	0.130	-0.075	-0.107	0.585	0.226	0.127	0.060	0.406	0.078
PKC-b-II_pS660	PRKCB	0.584	0.580	0.374	-0.697	-0.761	-0.650	-0.180	-0.315	-0.289
PKC-delta_pS664	PRKCD	0.066	-0.015	-0.031	0.202	0.192	0.136	-0.197	-0.122	-0.118
PKM2	PKM2	-0.010	-0.077	-0.123	0.508	0.257	0.166	0.390	0.341	-0.155
PLC-gamma2_pY759	PLCG2	0.038	-0.105	0.000	-0.075	-0.029	-0.162	-0.111	-0.223	-0.050
PLK1	PLK1	0.053	-0.217	0.344	0.874	0.462	0.136	0.266	0.553	0.000
PMS2	PMS2	-0.067	0.100	0.000	-0.220	0.077	0.052	0.197	-0.281	0.326
Porin	VDAC1	-0.010	-0.033	0.104	0.008	-0.086	-0.087	-0.014	-0.330	0.000
PR	PGR	0.010	-0.101	0.000	-0.217	-0.157	-0.289	-0.384	-0.290	-0.174
PRAS40	AKT1S1	0.021	0.165	-0.043	0.356	0.149	0.151	-0.309	-0.022	0.000
PRAS40_pT246	AKT1S1	-0.045	-0.046	-0.045	0.142	0.021	-0.059	0.266	0.149	0.009
PREX1	PREX1	-0.158	-0.209	-0.011	0.190	0.221	0.136	-0.341	-0.207	0.054
PTEN	PTEN	0.141	0.169	0.000	-0.220	-0.308	-0.047	-0.010	-0.126	-0.087
Puma	BBC3	0.003	0.078	0.000	-0.292	0.072	0.014	0.037	0.038	-0.065
PYGM	PYGM	0.000	0.124	-0.113	-0.449	-0.436	-0.440	0.115	-0.112	0.068
Rab11	RAB11A	0.000	-0.032	0.094	-0.335	-0.049	-0.214	-0.204	-0.051	-0.025
Rab25	RAB25	0.108	0.150	-0.004	0.143	0.109	0.136	-0.087	-0.301	-0.178
Rad50	RAD50	0.000	0.062	-0.169	0.100	-0.172	-0.191	-0.508	-0.221	0.500
Rad51	RAD51	-0.063	-0.095	0.000	0.045	0.106	0.123	0.106	-0.099	0.013
Raptor	RPTOR	0.090	-0.078	0.000	-0.364	-0.416	-0.428	-0.392	-0.176	-0.010
RBM15	RBM15	0.222	0.059	0.000	0.429	-0.188	-0.309	-0.179	0.108	-0.210
Rb_pS807_S811	RB1	0.000	-0.089	0.087	0.874	0.257	0.136	0.266	0.553	0.000
Rheb	RHEB	-0.153	0.081	0.000	-0.456	-0.119	0.127	-0.077	0.141	0.009
Rictor	RICTOR	0.260	0.523	0.177	-0.253	-0.059	-0.121	-0.112	-0.226	-0.319
Rictor_pT1135	RICTOR	0.048	0.040	0.000	0.000	-0.124	-0.264	-0.160	-0.163	-0.057

Rock-1	ROCK1	0.038	0.167	0.000	0.025	0.041	0.002	0.026	-0.193	-0.206
RPA32	RPA32	0.230	0.020	0.000	-0.544	-0.606	-0.531	-0.215	-0.296	-0.083
RPA32_pS4_S8	RPA32	0.000	0.084	0.014	-0.012	-0.181	-0.069	-0.030	-0.116	-0.222
RSK	RPS6KA1	0.062	0.008	-0.030	0.300	0.113	0.136	0.206	0.208	-0.141
S6	RPS6	0.065	0.113	0.000	-0.736	-0.678	-0.768	-0.206	-0.390	-0.182
S6_pS235_S236	RPS6	0.000	-0.017	0.420	0.646	0.029	-0.092	0.037	0.325	-0.037
S6_pS240_S244	RPS6	0.000	0.086	0.392	0.169	-0.324	-0.569	-0.439	-0.152	-0.627
SCD	SCD	-0.018	0.217	-0.115	0.171	0.084	0.000	0.278	0.225	0.000
SDHA	SDHA	-0.278	-0.341	-0.303	0.277	0.317	0.136	0.121	0.233	0.239
SF2	SRSF1	0.019	-0.100	0.000	-0.294	-0.009	0.011	-0.377	-0.240	-0.373
Shc_pY317	SHC1	-0.174	-0.158	0.043	0.301	0.117	-0.127	0.023	-0.384	0.000
SHP-2_pY542	PTPN11	0.098	-0.089	-0.009	-0.317	0.148	-0.002	-0.361	-0.305	0.000
SLC1A5	SLC1A5	0.000	0.429	-0.113	0.761	0.144	0.027	0.153	0.440	0.540
Smad1	SMAD1	0.000	0.029	0.077	0.089	0.082	0.041	0.046	-0.169	-0.047
Smad3	SMAD3	0.000	-0.204	0.021	-0.015	0.150	0.018	-0.001	0.208	-0.029
Smad4	SMAD4	0.000	-0.117	-0.079	-0.071	0.057	-0.018	0.225	0.186	0.115
SOD2	SOD2	-0.288	-0.217	-0.360	0.028	0.098	0.040	0.317	0.171	0.112
Sox2	SOX2	0.112	-0.150	-0.063	0.080	-0.005	-0.029	-0.274	0.113	0.000
Src	SRC	-0.197	0.214	0.000	0.236	0.223	0.184	-0.255	-0.130	0.133
Src_pY416	SRC	0.412	0.043	0.107	0.498	-0.136	0.125	-0.032	0.109	0.000
Src_pY527	SRC	-0.091	0.033	-0.060	0.773	0.307	0.441	-0.021	-0.108	0.046
Stat3	STAT3	0.637	0.630	0.096	-0.882	-1.499	-1.447	-1.172	-1.203	-0.990
Stat3_pY705	STAT3	0.123	0.296	0.000	-0.006	-0.117	-0.214	-0.350	-0.234	-0.301
Stat5a	STAT5A	-0.029	-0.006	0.038	-0.220	-0.141	-0.190	-0.308	-0.269	0.000
Stathmin-1	STMN1	-0.060	-0.115	-0.089	0.165	0.169	0.110	0.266	0.314	0.133
Syk	SYK	0.000	0.034	-0.109	0.702	0.161	0.083	0.093	0.381	0.050
Tau	MAPT	0.012	0.064	-0.044	-0.137	-0.027	-0.003	-0.115	-0.172	0.000
TAZ	TAZ	0.013	-0.124	0.000	0.118	-0.081	-0.179	-0.325	-0.203	-0.305
TFAM	TFAM	0.047	-0.191	-0.071	0.803	0.193	0.136	0.194	0.482	-0.030
TFRC	TFRC	-0.030	-0.016	0.229	0.842	0.264	0.104	0.234	0.543	0.000
TIGAR	TIGAR	-0.023	-0.103	-0.001	0.135	0.275	0.172	0.188	0.308	0.000
Transglutaminase	TGM2	-0.018	-0.172	-0.009	-0.291	-0.339	-0.448	0.288	0.484	0.817
TSC1	TSC1	0.233	0.350	0.109	-0.569	-0.583	-0.608	-0.286	-0.230	-0.349
TTF1	TTF1	-0.022	-0.217	0.038	-0.335	-0.166	-0.202	-0.292	-0.025	0.072
Tuberin	TSC2	0.000	-0.155	0.015	0.874	0.257	0.136	0.266	0.553	0.000
Tuberin_pT1462	TSC2	0.000	-0.058	-0.175	-0.197	-0.217	-0.258	-0.135	0.090	0.033
TWIST	TWIST1	0.000	-0.093	-0.014	0.005	0.156	-0.003	-0.173	0.032	0.087
Tyro3	TYRO3	0.126	0.118	-0.073	-0.077	0.135	0.068	-0.004	-0.074	0.000
UBAC1	UBAC1	0.000	-0.137	0.067	-0.120	-0.289	-0.285	-0.122	0.112	-0.231
Ubq-Histone-H2B	H2BFM	0.000	-0.104	-0.157	-0.164	0.037	-0.068	-0.265	-0.093	0.088
UGT1A	UGT1A	0.689	0.687	0.549	-0.240	-0.113	-0.257	-0.040	-0.096	0.000
VEGFR-2	KDR	0.349	0.449	0.000	-1.208	-1.082	-1.119	-1.127	-1.224	-0.526
VHL-EPPK1	VHL	0.000	0.000	0.200	0.738	0.121	0.097	0.273	0.509	-0.017
Vimentin	VIM	0.000	-0.029	0.155	-0.167	0.106	0.043	-0.326	-0.002	-0.049
Wee1	WEE1	-0.057	0.375	-0.371	-0.084	0.230	0.394	-0.031	-0.405	0.000
XBP-1	XBP1	0.000	-0.176	-0.065	-0.750	-0.221	-0.303	-0.244	-0.383	0.047
XIAP	XIAP	0.458	0.391	0.000	-0.232	-0.372	-0.219	-0.242	-0.314	-0.328
XPA	XPA	0.021	0.108	0.077	0.592	-0.013	0.039	0.127	0.271	-0.025
XPF	XPF	0.000	-0.047	-0.068	0.175	0.124	0.014	-0.518	0.300	0.057
XRCC1	XRCC1	0.000	-0.154	-0.110	-0.210	-0.124	-0.050	0.188	0.338	0.076
YAP	YAP1	0.000	-0.057	-0.105	0.105	0.036	-0.038	0.095	0.057	0.144
YAP_pS127	YAP1	0.000	-0.023	0.080	0.444	0.085	0.059	-0.111	0.123	-0.147
YB1	YBX1	0.150	-0.053	-0.155	0.356	-0.126	0.083	0.219	0.214	0.000
YB1_pS102	YBX1	0.020	0.386	0.000	-0.558	-0.372	-0.306	-0.190	-0.236	-0.089

Table S2. Top differentially activated/inhibited canonical pathways between NRD and NRI AMLs using Ingenuity Pathway Analysis (IPA).

Ingenuity Canonical Pathways	activation z-score	predicted regulation	p value
Production of Nitric Oxide and Reactive Oxygen Species in Macrophages	5.013	activated	2.455E-05
Role of NFAT in Regulation of the Immune Response	4.542	activated	5.248E-04
Tec Kinase Signaling	4.333	activated	5.888E-06
CD28 Signaling in T Helper Cells	4.041	activated	8.128E-04
IL-8 Signaling	3.857	activated	1.862E-06
Leukocyte Extravasation Signaling	3.742	activated	1.318E-10
B Cell Receptor Signaling	3.507	activated	2.089E-05
Signaling by Rho Family GTPases	3.464	activated	7.586E-05
Fc Receptor-mediated Phagocytosis in Macrophages and Monocytes	3.307	activated	6.026E-07
Rac Signaling	3.286	activated	2.692E-05
IL-6 Signaling	3.182	activated	1.148E-04
Actin Cytoskeleton Signaling	3.015	activated	7.244E-04
Dendritic Cell Maturation	2.967	activated	7.079E-05
ERK/MAPK Signaling	2.949	activated	2.818E-04
Phospholipase C Signaling	2.777	activated	2.042E-04
p38 MAPK Signaling	2.414	activated	3.162E-04
LPS/IL-1 Mediated Inhibition of RXR Function	2.400	activated	5.495E-05
Type II Diabetes Mellitus Signaling	2.294	activated	2.692E-04
Sphingosine-1-phosphate Signaling	2.263	activated	1.122E-05
Ephrin Receptor Signaling	2.263	activated	3.890E-04
Growth Hormone Signaling	2.236	activated	9.120E-05
Fc Epsilon RI Signaling	2.117	activated	8.128E-04
NF-kB Signaling	2.111	activated	1.479E-04
RhoGDI Signaling	-2.466	inhibited	8.318E-05
PI3K Signaling in B Lymphocytes	-3.087	inhibited	2.570E-05

Table S3. Top Ingenuity Pathway Analysis (IPA) predicted upstream regulators of differentially expressed genes.

upstream regulator	predicted regulation	activation z-score	p value
CDKN2A	activated	3.834	2.110E-10
SMARCA4	activated	2.938	3.180E-09
RBL1	activated	2.900	1.760E-05
HOXA9	activated	2.865	5.420E-07
CEBPE	activated	2.848	2.090E-13
RB1	activated	2.683	3.570E-06
ETS1	activated	2.635	9.490E-10
NFKB1	activated	2.603	2.000E-04
HMGA1	activated	2.603	6.590E-04
Gm21596/Hmgb1	activated	2.400	1.660E-02
CEBPA	activated	2.307	2.510E-12
EGR2	activated	2.216	1.730E-02
ING1	activated	2.213	3.890E-02
SPI1	activated	2.195	1.080E-21
BACH1	activated	2.152	3.890E-02
CEBPD	activated	2.101	1.130E-04
STAT3	activated	2.084	1.340E-10
ERG	activated	2.082	4.000E-12
TAF4	activated	2.043	5.210E-03
Msx3	inhibited	-2.000	4.440E-03
MITF	inhibited	-2.286	2.280E-08
PPARGC1A	inhibited	-2.319	1.350E-02
KLF15	inhibited	-2.579	3.530E-02
GFI1	inhibited	-2.906	4.180E-05
RUNX3	inhibited	-2.929	7.600E-05
NFE2	inhibited	-2.949	1.040E-06
GATA1	inhibited	-3.009	7.920E-24
SRF	inhibited	-3.141	2.960E-19
TBX2	inhibited	-3.162	1.440E-04
E2F3	inhibited	-3.289	3.790E-07
E2F1	inhibited	-4.151	3.790E-08
MKL2	inhibited	-4.459	3.880E-31
MKL1	inhibited	-4.631	1.780E-25

Table S4. Top differentially expressed transcriptional regulators between NRD and NRI AMLs.

	log2 expression values										
Gene	NRD1_1	NRD1_2	NRD1_3	NRI1_1	NRI1_2	NRI1_3	NRI2_1	NRI2_2	NRI2_3	NRD vs NRI fold change	corrected q-value
**up**											
GFI1	2.781	2.396	2.463	4.736	5.446	5.464	5.426	5.276	5.288	6.615	3.250E-04
CEBPE	5.441	5.259	5.136	7.234	7.971	7.819	7.447	7.535	7.277	4.819	3.250E-04
ID1	3.156	3.328	3.001	4.764	5.002	5.204	4.817	4.909	4.834	3.387	3.250E-04
DMRTB1	0.729	0.595	1.063	1.955	2.606	2.567	2.557	2.466	2.324	3.068	3.250E-04
TFEC	2.898	3.199	2.940	4.194	4.595	4.664	4.759	4.588	4.725	2.980	3.250E-04
CERS6	2.852	1.684	2.418	3.670	3.575	3.443	4.185	3.877	4.149	2.825	1.410E-03
LMO1	3.094	3.198	3.173	4.327	4.831	4.903	4.552	4.722	4.566	2.819	3.250E-04
MYB	4.706	4.564	4.584	5.757	6.139	6.109	6.168	6.069	6.174	2.735	3.250E-04
DACH1	2.415	1.722	2.037	3.175	3.535	3.456	3.591	3.549	3.618	2.693	3.250E-04
ARID3A	3.845	3.396	3.553	4.867	5.192	4.937	5.093	5.039	4.975	2.674	3.250E-04
**down**											
PAWR	3.358	3.393	3.267	2.337	1.993	2.223	1.788	2.116	1.991	0.416	3.250E-04
SOX12	3.001	3.140	2.976	2.008	1.615	1.881	1.638	1.747	1.540	0.406	3.250E-04
TFDP2	6.280	5.821	6.091	4.664	4.697	4.498	4.613	5.071	4.951	0.402	3.250E-04
ZFPM1	7.682	7.690	7.670	6.751	6.537	6.521	5.794	6.306	6.117	0.394	3.250E-04
KLF1	8.143	8.537	8.267	7.366	7.118	7.264	6.443	7.022	6.594	0.393	3.250E-04
CCNE1	7.001	7.363	7.210	6.162	5.645	6.112	5.343	5.795	5.424	0.368	3.250E-04
GATA1	7.214	7.479	7.290	6.226	5.917	6.158	5.438	5.912	5.606	0.366	3.250E-04
SOX6	5.323	4.545	5.066	3.733	3.305	3.145	3.260	3.866	3.683	0.359	3.250E-04
ZBTB32	4.207	4.288	4.276	2.741	2.335	2.534	2.572	3.053	2.777	0.333	3.250E-04
GFI1B	6.607	6.860	6.858	5.371	5.091	5.473	4.722	5.133	4.892	0.316	3.250E-04

Table S5. Clinical, cytogenetic, and molecular characteristics of primary AML patient samples.

ID	status	blasts	diagnostic karyotype	mutation(s)
AML1	de novo	87%	+6	FLT3-ITD
AML2	de novo	87%	normal	NPM1, IDH2(R140W)
AML4	de novo	92%	del(20)	IDH1(R132H)
AML5	de novo	65%	normal	FLT3-ITD
AML8	relapse	70%	t(1;3),del(5),-7,idem,t(1;17)	KRAS(Q61K),KRAS(Q61H)
